# Gut microbiota and osteoarthritis: mechanisms and translation

**DOI:** 10.3389/fimmu.2026.1873110

**Published:** 2026-07-08

**Authors:** Xiaoyuan Tian, Zhenan Qu, Ying Cao, Yichen Wang, Bocheng Zhang

**Affiliations:** 1 Second Affiliated Hospital, Dalian Medical University, Dalian, Liaoning, China; 2 Affiliated Zhongshan Hospital of Dalian University, Dalian, Liaoning, China; 3 School of Shuren International, Shenyang Medical College, Shenyang, Liaoning, China

**Keywords:** gut microbiota, gut–joint axis, immunity, microbial metabolites, osteoarthritis, precision medicine, probiotics

## Abstract

Osteoarthritis (OA) is increasingly recognised as a whole-joint disease driven by biomechanical stress, metabolic dysfunction, low-grade inflammation and immune dysregulation, yet effective disease-modifying treatments remain unavailable. Growing evidence suggests that gut microbiota dysbiosis may contribute to OA pathogenesis, giving rise to the concept of a functional and potentially targetable gut–joint axis. In this narrative review, we synthesise current evidence linking gut microbial alterations to OA and highlight the immunological mechanisms through which intestinal dysbiosis may influence joint degeneration. Human studies have identified OA-associated changes in gut microbial composition and microbial metabolites, whereas preclinical models, germ-free experiments and faecal microbiota transplantation studies provide mechanistic support for a contributory role of dysbiosis in cartilage damage, synovitis and subchondral bone remodelling. Gut dysbiosis can impair intestinal barrier integrity, facilitate systemic exposure to microbial products such as lipopolysaccharide, disturb short-chain fatty acid, bile acid and tryptophan-derived metabolite profiles, and alter enteroendocrine and immune signalling. These processes may activate Toll-like receptor, NF-κB, NLRP3 inflammasome, aryl hydrocarbon receptor and JAK/STAT pathways, thereby reshaping macrophage polarisation, Th17/Treg balance, mucosal IgA responses, innate lymphoid cell and γδT-cell activity, immunosenescence and low-grade systemic inflammation. Through these interconnected immune-metabolic pathways, the gut microbiota may influence cartilage catabolism, synovial inflammation, subchondral bone remodelling and inflammation-related pain. Microbiome-derived taxa, metabolites and host–microbe immune signatures might support risk assessment, endotype stratification and therapeutic monitoring; however, causality in humans remains incompletely established, and current findings are limited by heterogeneity in OA phenotypes, microbiome methods, host metabolic status and clinical endpoints. Microbiota-targeted strategies remain promising adjuncts rather than established disease-modifying treatments. Future studies should integrate standardised microbiome profiling, immune phenotyping, multi-omics approaches, longitudinal cohorts and rigorously designed clinical trials to translate gut–joint axis biology into microbiome-informed precision care for OA.

## 1 Introduction

Osteoarthritis (OA) is the most common chronic joint disease and a major cause of pain, disability and loss of mobility in ageing populations. It involves progressive cartilage degradation, subchondral bone remodelling, synovial inflammation, osteophyte formation and changes in periarticular tissues. The traditional view of OA as age-related mechanical wear is now insufficient. OA is better understood as a whole-joint disorder shaped by biomechanical stress, low-grade systemic inflammation, metabolic dysfunction, innate immune activation and altered pain processing (1, 2). This shift has important therapeutic implications. Current treatments mainly relieve symptoms through education, exercise, weight reduction, analgesics, intra-articular injections and joint replacement in advanced disease, whereas effective disease-modifying therapies capable of preventing structural progression remain unavailable. Identifying upstream, modifiable mechanisms that connect systemic risk factors with local joint degeneration is therefore essential.

The gut microbiota has emerged as one such potential regulator. As a complex microbial ecosystem, the intestinal microbiota participates in nutrient metabolism, epithelial barrier maintenance, mucosal immune education and systemic inflammatory control (3–5). Dysbiosis has been associated with obesity, insulin resistance, diabetes, ageing-related inflammation, rheumatoid arthritis, osteoporosis and other immune-metabolic disorders that overlap with recognised OA risk factors (6–9). These observations have led to the concept of a gut–joint axis, in which intestinal microbes, microbial products and microbial metabolites influence distant joint tissues by reshaping systemic immune and metabolic homeostasis (10–12). Recent human studies have reported associations between OA and altered gut microbial composition, microbial diversity and metabolite profiles, whereas animal models, germ-free experiments and faecal microbiota transplantation studies provide mechanistic evidence that intestinal microbial communities can modify cartilage damage, synovitis and subchondral bone remodelling (13, 14). Nevertheless, the current evidence remains heterogeneous, and microbiome alterations should be interpreted as context-dependent microbial and metabolic patterns rather than as a universal OA signature.

A central unresolved question is how intestinal dysbiosis is translated into joint pathology. Accumulating evidence suggests that this process is not mediated by a single linear pathway, but by interconnected immune-metabolic networks. Dysbiosis may influence OA through immune-metabolic pathways that connect intestinal barrier function, microbial metabolites and systemic inflammation (11, 15–25). Thus, the gut–joint axis should be viewed not only as a metabolic connection between intestine and joint, but also as an immune-mediated host–microbe communication network that may contribute to synovial activation, cartilage catabolism, subchondral bone remodelling and inflammation-related pain.

Beyond mechanism, the gut microbiota could support diagnosis, stratification and therapeutic monitoring. Microbial taxa, microbial genes and gut-derived metabolites detected in stool, serum or synovial fluid have been proposed as biomarkers of OA risk, severity and progression (13, 26–28). Multi-omics approaches that combine microbiome profiling with metabolomics, transcriptomics, proteomics, imaging features and clinical variables may improve stratification and identify inflammatory or metabolic OA phenotypes more likely to respond to microbiota-targeted interventions (29–31). Therapeutically, probiotics, prebiotics, synbiotics, dietary modulation, faecal microbiota transplantation (FMT), microbial metabolites and natural products that reshape intestinal microbial ecology are being investigated as adjunctive strategies for OA management (32–34). Most evidence, however, remains preclinical or comes from small clinical studies. Clinical translation will require rigorous trials, standardised sampling and sequencing protocols, strain-specific validation, long-term safety assessment and a clear separation of correlation from causality.

In this narrative review, we evaluate the role of the gut microbiota in OA from mechanistic, diagnostic and therapeutic perspectives. We first summarise evidence linking dysbiosis with OA across human studies, animal models and causal-inference approaches. We then examine mechanisms that may underlie the gut-joint axis, including barrier dysfunction, microbial metabolites, immune-inflammatory networks, cartilage catabolism and subchondral bone remodelling. Finally, we assess microbiome-derived biomarkers and microbiota-targeted interventions, and outline the methodological, regulatory and clinical challenges that must be addressed before microbiome-informed strategies can enter precision OA care.

## 2 Evidence linking gut dysbiosis to OA

Evidence linking gut dysbiosis with OA now spans clinical microbiome studies, animal models, microbial transplantation experiments and causal-inference analyses. OA has often been treated as a local joint disease, yet major risk factors such as ageing, obesity, metabolic syndrome, insulin resistance, low-grade inflammation and sex hormone changes are also associated with altered gut microbial composition and function (35, 36). These shared contexts support the idea that the intestinal microbiota may regulate systemic inflammatory and metabolic states that influence cartilage, synovium and subchondral bone. The evidence still requires caution. Most human studies are observational, microbial signatures vary across cohorts, and causal microbial drivers remain difficult to distinguish from secondary changes related to diet, medication, body composition or disease-associated behaviour.

### 2.1 Human microbiome signatures in OA

Human studies provide the first layer of evidence linking gut dysbiosis to OA. Several investigations have reported altered gut microbial diversity and composition in patients with OA compared with healthy controls, suggesting that OA is associated with a distinct intestinal microbial ecology (37, 38). In symptomatic hand OA, changes in the relative abundance of specific bacterial taxa have been observed, including enrichment of potentially pro-inflammatory genera such as Bilophila and Desulfovibrio and depletion of taxa such as Roseburia, which are commonly associated with short-chain fatty acid production and anti-inflammatory functions (37). Functional analyses in the same disease context further indicate perturbations in amino acid, carbohydrate and lipid metabolic pathways, implying that microbial dysbiosis in OA is not limited to taxonomic change but also involves altered metabolic capacity (37).

Evidence from knee OA also supports a relationship between gut microbial profiles and disease-relevant phenotypes. Microbial taxa and microbial metabolic pathways have been associated with pain, physical function, inflammatory markers and structural disease features (31, 36, 39). In overweight individuals, a random forest model based on seven microbial genera, including Gemmiger, Klebsiella, Akkermansia and Prevotella, showed discriminatory potential for identifying individuals at risk of OA, suggesting that gut microbiome profiles might contribute to non-invasive risk stratification (39). Serum metabolomic studies have also reported OA-associated changes in microbial-related metabolites and correlations between specific bacterial species and circulating metabolic products, reinforcing the possibility that gut microbial activity is reflected in systemic biochemical signatures relevant to OA (31). Additional support for the gut–joint axis comes from studies detecting gut-related microbial signals beyond the intestine. In one knee OA study, Enterococcus faecium and Staphylococcus hominis were isolated from both stool and joint-fluid samples and identified by matrix-assisted laser desorption ionization–time-of-flight mass spectrometry rather than by sequencing-based community profiling (40). However, because this type of low-biomass joint-fluid analysis is highly vulnerable to contamination and the available report does not clearly describe negative extraction, reagent or sampling controls, these findings should be interpreted cautiously. Microbial DNA has also been reported in OA synovial fluid, but DNA detection does not establish bacterial viability, active colonisation or direct gut-to-joint migration (41). Therefore, current evidence is more conservatively interpreted as indicating that microbial products, fragments or low-level microbial signals may reach joint-associated compartments and contribute to innate immune activation, rather than proving viable bacterial translocation as a general mechanism in OA.

Despite these findings, a consistent “OA microbiome” has not yet been established. Reported changes differ across studies and may vary according to OA site, disease stage, obesity status, diet, sex, age, medication exposure and sequencing methodology. Hand OA, knee OA, obesity-associated OA and postmenopausal OA may each be associated with different microbial and metabolic patterns rather than one shared dysbiotic state (36, 37, 42, 43). Thus, current human evidence is best viewed as supporting the presence of microbiome-associated OA phenotypes, not a single diagnostic microbial signature applicable to all patients.

### 2.2 Evidence from animal models

Animal studies provide stronger mechanistic evidence because they allow manipulation of the gut microbiota before or during OA development. In spontaneous OA models, altered gut microbial diversity and specific bacterial taxa have been associated with disease status. For example, studies in rhesus monkeys with spontaneous OA showed a trend towards reduced microbial diversity and identified taxa such as Mollicutes and Coprobacillus as potential disease-associated microbial markers (44). Although such models retain biological complexity, they remain largely associative. More direct evidence has come from surgically or chemically induced OA models in which the microbiota is experimentally altered. In destabilization of the medial meniscus (DMM) models, which mimic key features of human post-traumatic OA by inducing joint instability, altered load distribution and progressive cartilage degeneration after meniscal injury, antibiotic-induced changes in gut microbial communities have been linked to reduced systemic lipopolysaccharide (LPS), tumour necrosis factor-α (TNF-α) and IL-6 levels, decreased matrix metalloproteinase-13 (MMP-13) expression and attenuation of cartilage degradation (45). These data suggest that gut-derived inflammatory signals can modulate the severity of joint degeneration. However, antibiotic experiments are difficult to interpret because broad-spectrum antibiotics do not selectively remove pathogenic taxa; they also disrupt beneficial bacteria, alter microbial metabolites and can have direct host effects independent of microbiota depletion.

High-fat diet and obesity-related OA models further connect microbial dysbiosis with metabolic inflammation and joint pathology. Diet-induced dysbiosis can increase intestinal permeability, raise circulating LPS levels and amplify synovial inflammation and cartilage catabolism (46). These findings are particularly relevant because obesity increases OA risk through both mechanical loading and systemic metabolic inflammation, including in non-weight-bearing joints (35, 36). The microbiota may therefore help explain why metabolic OA phenotypes cannot be fully accounted for by biomechanics alone. Interventional studies using probiotics, prebiotics and microbial metabolites provide additional support. In monoiodoacetate-induced OA models, probiotic administration has been associated with reductions in IL-1β, IL-6 and TNF-α in serum or synovial fluid, improved cartilage morphology and lower OA histological scores (47, 48). Some probiotic effects appear to involve suppression of TLR2, TLR4 and NF-κB signalling, as well as modulation of metabolites such as γ-aminobutyric acid and short-chain fatty acids (SCFAs) (49). Propionate and butyrate have also been reported to support barrier integrity, immune regulation and cartilage matrix homeostasis in experimental OA settings (50, 51). These studies strengthen the argument that gut microbial function, rather than taxonomic composition alone, influences OA-relevant tissue responses.

FMT studies provide some of the most compelling preclinical evidence for a causal contribution of microbial communities. In antibiotic-depleted mice, FMT combined with probiotic administration inhibited DMM-induced cartilage damage and improved subchondral bone microarchitecture (52). In germ-free mice, transplantation of microbiota from OA patients produced distinct effects depending on donor metabolic status. Microbiota from non-obese OA donors exacerbated cartilage degeneration and synovitis, whereas microbiota from obese OA donors showed different inflammatory and microbial features, including enrichment of taxa such as Lactobacillus and Oscillibacter that were inversely related to inflammatory markers (53). These findings suggest that microbial effects on OA depend strongly on donor phenotype and host metabolic context.

### 2.3 Causality, heterogeneity and confounding

Although animal models support biological plausibility, establishing causality in humans remains challenging. Cross-sectional microbiome studies cannot determine whether dysbiosis precedes OA, results from OA-related lifestyle changes or reflects confounding factors such as diet, physical inactivity, analgesic use, comorbidities or prior antibiotic exposure. Pain and reduced mobility may themselves alter diet, body weight and medication use, all of which can reshape the gut microbiome. Therefore, microbial differences observed in OA patients may represent causes, consequences or correlates of disease. Mendelian randomisation studies have attempted to address causality by using host genetic variants associated with microbial traits as instrumental variables. Some analyses have identified microbial taxa, including Methanobacteriaceae and Desulfovibrionales, as potentially causally associated with knee OA (54, 55). Other studies have implicated microbial functional pathways, such as enterobactin biosynthesis and diacylglycerol biosynthesis, in OA susceptibility (56). However, the conclusions are not fully consistent across analyses, and some studies do not support a robust causal association between gut microbiome features and OA risk (57). These discrepancies may reflect differences in microbiome genome-wide association datasets, population ancestry, taxonomic resolution, statistical thresholds, OA phenotype definitions and weak instrument bias.

Another important limitation is phenotype heterogeneity. OA is not a single disease entity but a syndrome comprising multiple endotypes, including mechanically driven OA, metabolic OA, inflammatory OA, post-traumatic OA and postmenopausal or ageing-associated OA. A microbial feature that is relevant to obesity-associated knee OA may not apply to erosive hand OA or trauma-induced OA. Sex also modifies the relationship between gut microbiota, metabolism and joint pathology. High-fat diet studies have shown sex-dependent differences in gut microbial beta diversity, metabolic dysfunction, synovial inflammation and osteophyte formation (43, 58, 59). These data suggest that future microbiome studies should be stratified by sex, metabolic status, OA site and disease stage rather than treating OA as a homogeneous outcome. Technical heterogeneity further complicates interpretation. Differences in stool collection, storage, DNA extraction, 16S rRNA versus shotgun metagenomic sequencing, taxonomic databases, batch correction and statistical pipelines can produce divergent microbial profiles. High inter-individual variability in gut microbiota composition also makes it difficult to identify reproducible biomarkers across cohorts (60, 61). Longitudinal sampling, standardised protocols and multi-omics integration are therefore essential for distinguishing stable disease-associated signals from transient or method-dependent variation.

Taken together, the available evidence supports a biologically credible link between gut dysbiosis and OA, but the strength of evidence varies by study type. Human studies show association and biomarker potential; animal models and FMT experiments provide mechanistic and causal support; Mendelian randomisation offers suggestive but not definitive human causal inference. The main lines of evidence linking gut microbial dysbiosis to OA are summarised in **Table 1**. Future work should prioritise reproducible microbial functions and host-response pathways rather than isolated taxonomic differences. Such work will be essential before gut microbiota profiling can be used reliably for risk prediction, patient stratification or targeted intervention in OA.

**Table 1:** Evidence linking gut microbiota to osteoarthritis.

Evidence source	OA phenotype or model	Main findings	Evidence appraisal	References
Human cross-sectional microbiome studies	Clinical OA cohorts, mainly hand or knee OA	OA has been associated with altered gut microbial diversity and composition, including enrichment of potentially pro-inflammatory taxa and depletion of bacteria linked to anti-inflammatory metabolite production.	Directly relevant to human disease, but mostly observational and sensitive to diet, medication, obesity, age, sex and sequencing methods.	(37, 38, 41, 54)
Symptomatic hand OA cohorts	Symptomatic hand OA	Increased Bilophila and Desulfovibrio and reduced Roseburia have been linked to altered amino acid, carbohydrate and lipid metabolic pathways.	Provides phenotype-specific human evidence, but requires independent validation and does not establish causality.	(37, 103, 104)
Knee OA and obesity or metabolic OA studies	Knee OA, overweight or obesity-associated OA, metabolic OA phenotypes	Microbial taxa and pathways have been associated with pain, physical function, inflammatory markers and structural features.	Supports metabolic stratification, but microbiome effects are difficult to separate from loading, diet, weight loss and comorbidity.	(31, 36, 39, 62, 119)
Synovial fluid or microbial DNA studies	Predominantly knee OA	Gut-related microbial signals, including taxa also detected in stool, have been reported in synovial fluid or joint-associated compartments.	Supports biological plausibility for gut-joint communication, but low-biomass contamination and technical artefacts remain important concerns.	(27, 40, 41)
Animal OA models	DMM, MIA, high-fat-diet-induced, spontaneous and postmenopausal-like OA models	Experimental dysbiosis has been linked to synovial inflammation, cartilage catabolism, subchondral bone remodelling and systemic inflammatory mediators.	Allows controlled mechanistic testing, but model-specific findings may not fully reproduce slow and heterogeneous human OA.	(44–46, 59)
Antibiotic-mediated microbiota depletion	Mainly mouse OA models, including DMM and metabolic or ovariectomy-associated settings	Microbiota depletion has been associated with reduced LPS, TNF-α, IL-6 and MMP-13 levels and attenuated cartilage degradation in some models.	Shows that microbiota alteration can modify OA-relevant outcomes, but antibiotics are non-specific and unsuitable for long-term OA therapy.	(45, 77, 107)
Germ-free and FMT experiments	Recipient animals exposed to OA-associated or intervention-modified microbiota	Donor-dependent microbial communities can modify cartilage damage, synovitis, subchondral bone architecture and inflammatory features.	Provides stronger causal support than association studies, but effects depend on donor phenotype, recipient model and transfer protocol.	(52, 53, 129)
Mendelian randomisation and causal-inference studies	Human OA risk or OA-related traits	Candidate microbial taxa or features have been nominated as potentially related to OA susceptibility, although findings vary across analyses.	Useful for prioritising mechanisms, but dependent on instrument validity, ancestry, taxonomic resolution and causal assumptions.	(55–57)

DMM, destabilization of the medial meniscus; FMT, faecal microbiota transplantation; IL-6, interleukin-6; LPS, lipopolysaccharide; MIA, monoiodoacetate; MMP-13, matrix metalloproteinase-13; OA, osteoarthritis; TNF-α, tumour necrosis factor-alpha.

## 3 Biological mechanisms of the gut–joint axis

**Figure 1:**
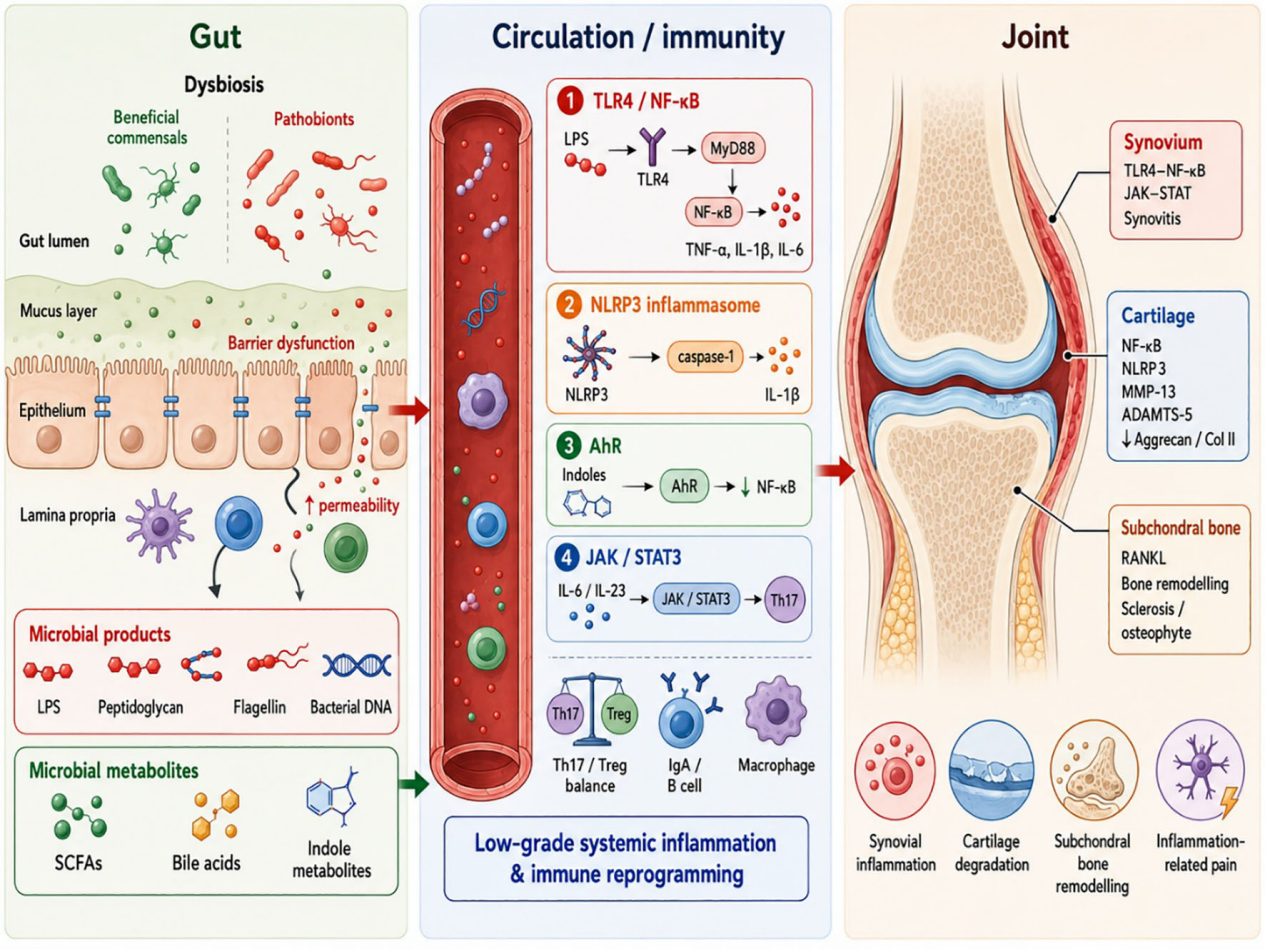
The gut–joint axis in osteoarthritis. Gut dysbiosis may contribute to osteoarthritis through a three-step gut–circulation–joint pathway. In the gut, loss of beneficial commensals and enrichment of pathobionts impair epithelial barrier integrity and increase intestinal permeability, allowing microbial products such as LPS, peptidoglycan, flagellin and bacterial DNA to enter the circulation. At the same time, altered microbial metabolism changes the levels of SCFAs, bile acids and tryptophan-derived metabolites. These gut-derived signals reshape systemic immune responses through TLR/NF-κB, NLRP3 inflammasome, AhR and JAK/STAT-related pathways, promoting low-grade inflammation and immune dysregulation. In the joint, these immune-metabolic changes may drive synovial inflammation, chondrocyte dysfunction, cartilage matrix degradation, subchondral bone remodelling and pain-related inflammatory signalling. The pathways shown represent proposed mechanisms linking gut microbial dysbiosis to OA progression. ADAMTS-5, a disintegrin and metalloproteinase with thrombospondin motifs 5; AhR, aryl hydrocarbon receptor; Col II, type II collagen; DNA, deoxyribonucleic acid; IgA, immunoglobulin A; IL, interleukin; JAK/STAT, Janus kinase/signal transducer and activator of transcription; LPS, lipopolysaccharide; MMP-13, matrix metalloproteinase-13; MyD88, myeloid differentiation primary response 88; NF-κB, nuclear factor-κB; NLRP3, NOD-, LRR- and pyrin domain-containing protein 3; OA, osteoarthritis; RANKL, receptor activator of nuclear factor-κB ligand; SCFAs, short-chain fatty acids; Th17, T helper 17 cell; TLR4, Toll-like receptor 4; TNF-α, tumour necrosis factor-α; Treg, regulatory T cell.

The gut–joint axis explains how intestinal microbial communities may influence distant joint tissues (**Figure 1**). We organise the gut–joint axis into four mechanistic levels: intestinal barrier dysfunction and microbial translocation, microbial metabolite signalling, immune-cell reprogramming, and osteochondral tissue remodelling. These pathways are not independent; rather, they interact across the gut, circulation and joint microenvironment to influence synovial inflammation, cartilage catabolism, subchondral bone remodelling and pain-related inflammatory signalling. The following sections therefore emphasise mechanistic connections rather than reiterating causal evidence from animal models or faecal microbiota transplantation studies.

### 3.1 Barrier dysfunction and microbial translocation

A central mechanism linking gut dysbiosis to OA is the disruption of intestinal barrier integrity. Under physiological conditions, the intestinal epithelium, mucus layer, antimicrobial peptides and tight junction proteins restrict the passage of microbial products into the systemic circulation. Dysbiosis can weaken this barrier by reducing beneficial commensals, impairing tight junction proteins such as zonula occludens-1 and occludin, and altering epithelial immune responses. This state of increased intestinal permeability, commonly referred to as “leaky gut”, enables microbial components to enter the circulation and promote chronic low-grade inflammation (40, 62, 63).

LPS, a major component of the outer membrane of Gram-negative bacteria, is one of the best-characterised microbial products implicated in OA. Increased circulating LPS has been observed in OA-related experimental models and has been associated with systemic inflammatory activation (46, 64). LPS can bind to lipopolysaccharide-binding protein and activate Toll-like receptor 4 (TLR4) signalling on immune cells, synovial cells and chondrocytes. Subsequent activation of MyD88-dependent pathways and NF-κB signalling induces the production of inflammatory cytokines, chemokines and catabolic enzymes, including TNF-α, IL-1β, IL-6 and MMP-13. These mediators promote cartilage extracellular matrix degradation, synovial inflammation and osteochondral tissue remodelling (45, 46, 64).

Experimental studies provide mechanistic support for this pathway. In diet-induced or surgically induced OA models, gut dysbiosis can increase intestinal permeability and circulating LPS, thereby intensifying synovitis and cartilage degeneration (46). Conversely, interventions that reduce endotoxaemia or restore barrier function, including selected probiotics, dietary fibre and microbial metabolites, can attenuate inflammatory responses and improve OA-related structural changes (34, 51, 65). These observations suggest that the intestinal barrier functions as a biological checkpoint: when intact, it limits systemic exposure to microbial inflammatory signals; when disrupted, it allows gut-derived molecules to amplify joint inflammation.

Microbial translocation may also extend beyond soluble microbial products. Low-level microbial signals have been detected in joint-associated compartments, but these findings require cautious interpretation because synovial fluid is a low-biomass sample (40, 41). These findings suggest that intestinal microbial signals may reach joint-associated compartments, either through direct microbial translocation, microbial fragments or circulating immune complexes. However, detection of microbial DNA does not necessarily indicate viable bacteria or infection. In OA, the more plausible mechanism is that microbial products or fragments provide persistent innate immune stimulation that lowers the threshold for synovial activation and cartilage catabolism.

### 3.2 Microbial metabolites as molecular mediators

Microbial metabolites represent a second major route through which the gut microbiota influences joint tissues. These metabolites are biologically active molecules generated by microbial fermentation, amino acid metabolism, bile acid transformation and lipid metabolism. They can enter the circulation, interact with host receptors and regulate epithelial, immune and skeletal cell function. In OA, microbial metabolites may be protective or deleterious depending on their concentration, tissue context and host metabolic state.

SCFAs, particularly acetate, propionate and butyrate, are among the most extensively studied protective microbial metabolites. Produced through fermentation of dietary fibre, SCFAs help maintain intestinal barrier integrity, regulate immune tolerance and modulate inflammatory signalling. In OA models, SCFAs have been associated with preservation of cartilage and subchondral bone integrity, suppression of inflammatory cytokines and improvement of extracellular matrix homeostasis (50, 51, 66). Sustained-release butyrate administration in patients with OA has been reported to modulate immune responses, including reduction of pro-inflammatory T helper-cell activation and improvement of the Th17/Treg balance (50). Propionate treatment in OA rats attenuated disease progression by restoring gut microbial balance, enhancing barrier function and regulating chondrocyte gene expression linked to matrix maintenance (51). At the cellular level, SCFAs may affect chondrocytes through several mechanisms. Butyrate can act as a histone deacetylase inhibitor and thereby influence gene transcription, inflammatory responses and cellular stress pathways. Propionate and butyrate may also reduce oxidative stress, suppress NF-κB activation, improve mitochondrial homeostasis and regulate autophagy. These effects are relevant because chondrocyte senescence, impaired autophagy and excessive oxidative stress contribute to cartilage matrix breakdown in OA. SCFA-producing bacteria, including taxa related to Roseburia, Faecalibacterium and other anaerobic commensals, may therefore contribute to a metabolically favourable environment that protects the osteochondral unit (37, 51, 66).

Tryptophan-derived metabolites provide another important mechanistic link. Gut bacteria convert dietary tryptophan into indole derivatives, including indole-3-propionic acid and indole-3-aldehyde. These metabolites can activate the aryl hydrocarbon receptor (AhR), a ligand-regulated transcription factor involved in mucosal immunity, barrier integrity and inflammatory control. In chondrocytes, tryptophan-derived indole metabolites have been shown to suppress inflammatory responses through the AhR–NF-κB axis, reducing the expression of pro-inflammatory cytokines and matrix-degrading enzymes while supporting anabolic markers such as aggrecan and type II collagen (17, 67). However, independent evidence remains limited, and the biological role of tryptophan–AhR signalling in OA appears to be context-dependent. Clinical metabolomic data have linked circulating tryptophan metabolites with erosive hand OA and pain (68), whereas experimental work has suggested that activated microbiome-associated tryptophan metabolism and AhR upregulation may also promote OA progression in some settings (69). These findings indicate that tryptophan-derived metabolites should be interpreted as modulators of AhR-dependent immune-cartilage crosstalk rather than uniformly protective mediators. This pathway illustrates how microbial metabolism can directly influence chondrocyte phenotype and cartilage matrix balance. Bile acid metabolism is also increasingly recognised as part of the gut–joint axis. Primary bile acids synthesised by the host are converted by intestinal microbes into secondary bile acids, which regulate metabolism and immunity through receptors such as FXR and TGR5. Dysregulated bile acid profiles, including alterations in deoxycholic acid, have been associated with symptomatic hand OA and may interact with host genetic variation in bile acid receptor pathways (16, 70). Some microbiota-modulating interventions, including plant-derived compounds, appear to alleviate OA partly by restoring bile acid metabolism and reducing inflammatory macrophage polarisation (70). These findings suggest that bile acids are not merely digestive molecules but systemic immunometabolic mediators relevant to joint disease.

Other microbial or microbiota-modulated metabolites may affect chondrocyte survival, ferroptosis and matrix turnover. Urolithin A, a gut microbiota-derived metabolite, has been reported to protect against OA by activating the AMPK/mTOR/HIF-1α pathway and reducing chondrocyte ferroptosis and inflammation (71). Urolithin B may regulate iron homeostasis through the FGFR3/NCOA4/FTH1 axis, thereby limiting ferroptosis-associated cartilage degeneration (72). Capsiate has also been linked to reduced ferroptosis-dependent OA progression by modulating HIF-1α-related pathways (73). Together, these data suggest that the metabolic output of the gut microbiota can influence chondrocyte fate through inflammation, autophagy, oxidative stress, iron metabolism and extracellular matrix synthesis.

### 3.3 Inflammatory signalling pathways

Immune regulation is a key bridge between gut dysbiosis and joint pathology. The gut microbiota educates both innate and adaptive immunity, shaping macrophage function, T-cell differentiation, inflammasome activation and cytokine production. When microbial homeostasis is disturbed, immune tolerance can be replaced by chronic low-grade inflammation, which contributes to OA progression. Dysbiosis-associated microbial products and metabolites activate inflammatory signalling pathways that amplify OA-related tissue catabolism. The cellular mechanisms through which these immune alterations contribute to OA are discussed in Section 3.4.

Inflammasome activation also connects microbial signals with cartilage catabolism. NLRP3 inflammasome activation promotes maturation of IL-1β, a potent inducer of chondrocyte catabolic activity and MMP expression (74). Microbial products, oxidative stress and lipid mediators can all contribute to inflammasome priming or activation. Pterostilbene and other microbiota-modulating compounds have been reported to reduce OA progression by inhibiting NF-κB activation and the NLRP3 inflammasome while improving gut microbial composition (75). These data suggest that microbial dysbiosis may not simply increase cytokine abundance but can also regulate upstream inflammatory platforms that sustain OA pathology.

The downstream effector of many of these immune pathways is matrix degradation. MMP-13 is particularly important because it cleaves type II collagen, the principal collagen of articular cartilage. Inflammatory cytokines such as IL-1β, TNF-α and IL-6 can drive MMP-13 expression through NF-κB, MAPK and HIF-2α-related pathways (74, 76). Gut microbiota-targeted interventions that reduce systemic inflammation or suppress NF-κB/HIF-2α signalling have been associated with lower MMP-13 expression and reduced cartilage damage (76, 77). Thus, the microbiota may influence structural progression of OA by regulating the inflammatory signals that converge on cartilage-degrading enzymes.

### 3.4 Innate and adaptive immunity in the gut–joint axis

The gut–joint axis should be understood as an immune-mediated host–microbe communication network rather than a simple metabolic pathway. Under physiological conditions, the intestinal mucosa integrates epithelial cells, mucus, antimicrobial peptides, secretory IgA, dendritic cells, macrophages, T cells, B cells and innate lymphoid cells to maintain immune tolerance while preventing systemic exposure to microbial antigens (11, 12, 78, 79). Gut dysbiosis can disrupt this equilibrium by weakening epithelial barrier integrity, reducing tolerogenic microbial metabolites and increasing exposure to microbial-associated molecular patterns. These changes may shift mucosal and systemic immunity from a regulatory state towards chronic low-grade inflammation, thereby lowering the threshold for synovial activation, chondrocyte catabolism and subchondral bone remodelling (15, 21, 78, 80).

Innate immunity constitutes an important interface between the intestinal microbiota and joint tissues. Beyond initiating inflammatory signalling pathways, gut microbiota-derived products and metabolites shape the phenotype and function of multiple innate immune-cell populations. These effects influence tissue homeostasis, inflammatory resolution and susceptibility to chronic synovial inflammation. Macrophages are central effector cells linking microbial sensing to joint inflammation. In OA synovium, macrophage accumulation and activation are associated with synovitis, pain and cartilage degradation. A pro-inflammatory M1-like phenotype produces TNF-α, IL-1β, IL-6, nitric oxide and matrix-degrading mediators, whereas M2-like macrophages are more closely associated with inflammation resolution and tissue repair (81, 82). Gut-derived inflammatory signals, including LPS and altered bile acids, may favour M1 polarisation through TLR4/NF-κB and inflammasome-related pathways, whereas SCFAs, selected secondary bile acids and other microbial metabolites may promote anti-inflammatory macrophage responses (15, 16). Thus, macrophage polarisation represents a plausible cellular node through which dysbiosis can translate into synovial inflammation and osteochondral tissue damage.

Adaptive immune imbalance provides a second layer of gut–joint communication. Although OA is not a classical autoimmune disease, T cells are present in OA synovium and can contribute to chronic synovial inflammation (83, 84). The balance between Th17 cells and regulatory T cells is particularly relevant because both populations are strongly influenced by intestinal microbiota and microbial metabolites (19). Th17-skewed immunity can increase IL-17 production, which promotes IL-6, TNF-α, matrix metalloproteinases, aggrecanases and RANKL-mediated osteoclast activity, thereby linking mucosal immune skewing to cartilage matrix degradation and subchondral bone remodelling (85). By contrast, Treg cells maintain immune tolerance through IL-10, TGF-β and CTLA-4-dependent mechanisms and may limit excessive inflammation in both the gut and joint microenvironments. SCFAs can modulate T-cell differentiation through G-protein-coupled receptors and histone deacetylase inhibition, whereas tryptophan-derived indoles can activate AhR and influence IL-22, IL-10 and Th17-related pathways (17, 86, 87). These observations support a model in which loss of microbiota-derived regulatory signals favours systemic immune activation and OA-relevant tissue inflammation.

B cells and mucosal antibody responses are another underexplored component of the gut–joint axis. In the intestine, secretory IgA limits epithelial adhesion and penetration of microorganisms, shapes commensal communities and reduces systemic exposure to microbial antigens (23). Defects in IgA-mediated containment can increase exposure to commensal-derived antigens and promote systemic immune stimulation. In the joint, B cells are not usually dominant immune cells in OA, but they have been detected in synovial tissue and may participate in antigen presentation, cytokine production and local immune organisation (88, 89). Therefore, gut dysbiosis could theoretically influence OA not only through soluble inflammatory mediators but also through altered mucosal antibody selection and systemic antigenic load. However, direct evidence linking gut IgA responses to OA progression is still limited, and this pathway should be presented as a plausible but insufficiently validated mechanism.

Innate lymphoid cells and γδT cells provide additional links between microbial cues, barrier function and systemic inflammation. ILC3s are enriched at mucosal surfaces and produce IL-22, a cytokine that supports epithelial repair, mucus production, antimicrobial peptide expression and barrier integrity (89, 90). Microbiota-derived SCFAs and AhR ligands can promote IL-22 production by CD4+ T cells and ILCs through GPR41, HDAC inhibition, AhR, HIF-1α and STAT3-related mechanisms (90). Conversely, dysbiosis may weaken this barrier-protective circuit and facilitate microbial product translocation. γδT cells also reside in the intestinal epithelium and rapidly produce IL-17 and IL-22 in response to microbial and metabolic cues (22, 91). Experimental evidence indicates that SCFAs, particularly propionate, can reduce IL-17 and IL-22 production by intestinal γδT cells (22). Although OA-specific data on ILCs and γδT cells remain scarce, these populations are immunologically relevant because they connect gut microbial metabolism with IL-17/IL-22-dependent mucosal and systemic inflammatory programmes.

Ageing adds a further immunological dimension to the gut–joint axis. OA prevalence increases sharply with age, and ageing is accompanied by immunosenescence and inflammaging. Immunosenescence involves thymic involution, contraction of the naïve T-cell pool, accumulation of oligoclonal memory or senescent T cells, impaired immune resolution and altered macrophage and dendritic-cell function (24, 92). Inflammaging is characterised by chronic low-grade elevation of inflammatory mediators such as IL-6, TNF-α and acute-phase proteins, which overlap with cytokine networks implicated in OA (93, 94). Age-related intestinal barrier dysfunction and gut microbiota shifts may further amplify this systemic inflammatory state. In this context, dysbiosis may not act as an isolated trigger, but as a contributor to an ageing-associated immune environment that favours synovitis, cartilage catabolism, subchondral bone remodelling and pain sensitisation.

**Figure 2:**
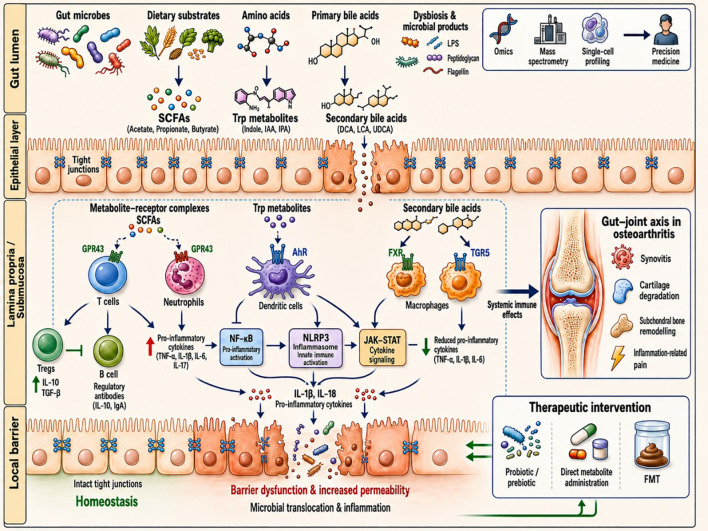
Immune signalling pathways linking gut dysbiosis to osteoarthritis. Gut dysbiosis alters microbial products and metabolites, including LPS, SCFAs, tryptophan metabolites and secondary bile acids. These signals act through GPR43, AhR, FXR/TGR5 and pattern-recognition pathways to regulate mucosal immune cells and activate NF-κB, NLRP3 inflammasome and JAK–STAT signalling. The resulting immune imbalance promotes barrier dysfunction, systemic inflammation and downstream joint changes, including synovitis, cartilage degradation, subchondral bone remodelling and inflammation-related pain. AhR, aryl hydrocarbon receptor; DCA, deoxycholic acid; FMT, faecal microbiota transplantation; FXR, farnesoid X receptor; GPR43, G-protein-coupled receptor 43; IgA, immunoglobulin A; IL, interleukin; IPA, indole-3-propionic acid; JAK/STAT, Janus kinase/signal transducer and activator of transcription; LCA, lithocholic acid; LPS, lipopolysaccharide; NF-κB, nuclear factor-κB; NLRP3, NOD-, LRR- and pyrin domain-containing protein 3; OA, osteoarthritis; SCFAs, short-chain fatty acids; TGF-β, transforming growth factor-β; TGR5, Takeda G-protein-coupled receptor 5; TNF-α, tumour necrosis factor-α; Treg, regulatory T cell; Trp, tryptophan; UDCA, ursodeoxycholic acid.

Taken together, innate and adaptive immunity provide the mechanistic bridge between gut dysbiosis and joint degeneration. Together, macrophages, T cells, B cells, ILCs and γδT cells provide cellular routes through which dysbiosis may reshape systemic and joint inflammation (**Figure 2**). The central immunological thesis of the gut–joint axis is therefore that dysbiosis promotes OA not merely by changing microbial composition, but by reprogramming mucosal, systemic and joint immune responses. Future studies should combine microbiome profiling with immune phenotyping, single-cell analysis and longitudinal sampling to determine which immune pathways are causal, which are secondary to joint damage and which are therapeutically targetable.

### 3.5 Subchondral bone, metabolism and sex differences

OA is a disease of the whole joint, and gut microbiota-mediated mechanisms extend beyond cartilage to subchondral bone, synovium, infrapatellar fat pad and systemic metabolism. Subchondral bone remodelling is a defining feature of OA and contributes to altered joint biomechanics, osteophyte formation, bone marrow lesions and pain. The gut microbiota may influence this process through regulation of calcium and phosphorus absorption, vitamin metabolism, osteoclastogenesis, osteoblast activity and systemic inflammatory tone (95–100).

SCFAs can modulate bone remodelling by regulating osteoclast and osteoblast function, while also improving mineral absorption and reducing inflammatory drivers of bone resorption (96, 98). Dysbiosis-associated increases in TNF-α and IL-6 may promote osteoclastogenesis and bone loss, whereas restoration of microbial balance can improve bone microarchitecture in experimental models (77, 96, 98). Although some of this evidence comes from osteoporosis models rather than OA-specific systems, it is relevant because subchondral bone turnover and cartilage degeneration are tightly coupled in OA. Microbial effects on bone should therefore be interpreted specifically in relation to the osteochondral unit rather than generalised skeletal metabolism.

Metabolic inflammation provides an additional link between gut microbiota and joint structural damage. Obesity and insulin resistance are associated with dysbiosis, increased gut permeability, endotoxaemia, adipose tissue inflammation and altered circulating metabolites (35, 36). These factors can promote synovial inflammation, infrapatellar fat pad activation, cartilage catabolism and subchondral bone abnormalities. The gut microbiota may therefore help explain why metabolic syndrome increases OA risk not only through mechanical loading but also through systemic inflammatory and metabolic pathways. In this context, the gut–joint axis is particularly relevant to metabolic OA, in which microbial and host metabolic disturbances converge on the joint.

Sex differences further shape gut microbiota-mediated OA mechanisms. Women have a higher prevalence of OA after midlife, and postmenopausal hormonal change is associated with altered immune responses, bone remodelling and metabolic risk. Estrogen deficiency can promote gut dysbiosis, increase intestinal permeability and enhance low-grade systemic inflammation (42). In ovariectomized models, microbiota modulation has been linked to changes in bone mineral density, cartilage injury, LPS and TNF-α, suggesting that estrogen–microbiota interactions may influence both skeletal and joint homeostasis (42, 77).

High-fat diet models show that the relationship between gut microbiota, metabolism and joint pathology differs by sex. Male and female mice exposed to high-fat diet exhibit distinct gut microbial beta diversity and tissue-level OA phenotypes. Males may develop more pronounced cartilage pathology and osteophyte formation, whereas females show greater synovial hyperplasia and upregulation of pro-inflammatory and pro-fibrotic genes in synovium and infrapatellar fat tissue (43). In rats exposed to high-fat/high-sucrose diets, males developed bone lesions more readily than females, and prebiotic fibre supplementation produced more marked microbiota effects in males (59). These findings emphasise that microbiota-mediated OA mechanisms are modified by sex hormones, metabolic state and tissue-specific responses.

Taken together, the biological mechanisms of the gut–joint axis are multifactorial and context-dependent. To improve clarity, we summarise gut-derived microbial products and metabolites separately from host immune, metabolic and tissue responses in **Tables 2**, **3**. Overall, dysbiosis may affect OA through barrier dysfunction, altered metabolites, immune reprogramming and osteochondral remodelling. These pathways converge on key OA processes, including synovitis, chondrocyte catabolism, extracellular matrix degradation, osteophyte formation and inflammation-related pain. Future mechanistic studies should move from broad taxonomic descriptions towards functional microbial pathways, metabolite–receptor interactions and host-response networks that define specific OA endotypes. Such mechanistic precision will be essential for translating gut microbiota research into targeted, safe and clinically meaningful interventions.

**Table 2:** Microbial products and metabolites implicated in the gut–joint axis in osteoarthritis.

Microbial product or metabolite	Main source or trigger	Principal receptors or pathways	Proposed OA-relevant effects	References
LPS and other microbial-associated molecular patterns	Gram-negative bacteria, dysbiosis and increased intestinal permeability	LBP–CD14–TLR4, MyD88, NF-κB and MAPK signalling	May promote systemic low-grade inflammation, synovial activation, chondrocyte catabolism and increased expression of TNF-α, IL-1β, IL-6 and MMP-13.	(45, 46, 62–64)
Microbial fragments and bacterial DNA	Barrier disruption, microbial translocation or low-level microbial signals detected outside the gut	Pattern-recognition receptors, including TLR-dependent pathways	May provide persistent innate immune stimulation in joint-associated compartments; however, low-biomass detection requires careful interpretation and contamination control.	(40, 41, 164)
Short-chain fatty acids, including acetate, propionate and butyrate	Fermentation of dietary fibre by commensal anaerobes	GPR41, GPR43, GPR109A and histone deacetylase inhibition	May strengthen epithelial barrier integrity, promote immune tolerance, modulate macrophage and T-cell responses, and support cartilage and subchondral bone homeostasis.	(15, 50, 51, 66, 102)
Tryptophan-derived indole metabolites	Microbial metabolism of dietary tryptophan	AhR, AhR–NF-κB crosstalk and IL-22-related pathways	May regulate mucosal immunity, barrier repair and chondrocyte inflammatory responses; current evidence suggests context-dependent protective or pathogenic effects depending on ligand, tissue and disease setting.	(17, 67–69, 103, 104, 163)
Microbiota-modulated bile acids	Microbial transformation of primary into secondary bile acids	FXR, TGR5 and macrophage-related metabolic signalling	May link dysbiosis with metabolic inflammation, macrophage polarisation and synovial or cartilage responses.	(16, 70)
Urolithins and other microbiota-modulated lipid-related metabolites	Microbial transformation of dietary polyphenols and host–microbial lipid metabolism	AMPK/mTOR/HIF-1α, FGFR3/NCOA4/FTH1 and ferroptosis-related pathways	May influence chondrocyte oxidative stress, autophagy, ferroptosis, matrix turnover and inflammatory signalling.	(71–73)

AhR, aryl hydrocarbon receptor; FXR, farnesoid X receptor; GPR, G-protein-coupled receptor; HIF-1α, hypoxia-inducible factor-1α; IL, interleukin; LBP, lipopolysaccharide-binding protein; LPS, lipopolysaccharide; MAPK, mitogen-activated protein kinase; MMP-13, matrix metalloproteinase-13; NF-κB, nuclear factor-κB; OA, osteoarthritis; TGR5, Takeda G-protein-coupled receptor 5; TLR, Toll-like receptor; TNF-α, tumour necrosis factor-α.

**Table 3:** Host immune, metabolic and tissue responses linking gut dysbiosis to osteoarthritis.

Host response or tissue pathway	Gut-related trigger	Main cellular or tissue targets	Proposed OA-relevant consequences	References
Intestinal barrier dysfunction and microbial translocation	Loss of beneficial commensals, impaired mucus layer and reduced tight-junction integrity	Intestinal epithelium, systemic circulation and joint-associated immune cells	May increase exposure to microbial products and amplify systemic low-grade inflammation relevant to synovitis and cartilage catabolism.	(40, 62, 63, 80)
Macrophage polarisation	LPS, altered bile acids, reduced SCFAs and inflammatory microbial signals	Synovial macrophages, monocytes and innate immune cells	May favour M1-like activation, increasing TNF-α, IL-1β, IL-6, nitric oxide and matrix-degrading mediators; anti-inflammatory microbial metabolites may support resolution-associated responses.	(15, 16, 70, 81, 82)
Th17/Treg imbalance	Reduced tolerogenic metabolites, altered SCFA and tryptophan metabolism, and persistent microbial antigen exposure	CD4+ T-cell subsets, synovium and osteochondral tissues	Th17-skewed responses may increase IL-17, IL-6, TNF-α, MMPs, aggrecanases and RANKL-mediated osteoclast activity, whereas Treg responses may limit excessive inflammation.	(17, 19, 50, 85–87)
B cells and mucosal IgA responses	Dysbiosis-associated disruption of mucosal immune containment	Secretory IgA, intestinal B cells, synovial B cells and antigen-presenting networks	Impaired IgA-mediated containment may increase systemic exposure to commensal antigens; the direct contribution of B-cell responses to OA progression remains insufficiently validated.	(23, 88, 89)
Innate lymphoid cells and γδT cells	Altered SCFAs, AhR ligands and epithelial barrier signals	ILC3s, γδT cells and IL-17/IL-22-related mucosal circuits	May regulate epithelial repair, antimicrobial peptide production and IL-17/IL-22-dependent inflammatory programmes, with potential downstream effects on systemic inflammation.	(21, 22, 90, 91)
NLRP3 inflammasome and cartilage matrix catabolism	Microbial products, oxidative stress, lipid mediators and inflammatory cytokines	Chondrocytes, synoviocytes and cartilage extracellular matrix	May increase IL-1β maturation, NF-κB/MAPK/HIF-2α signalling, MMP-13 expression and collagen degradation.	(74–77)
Subchondral bone and metabolic remodelling	Metabolic endotoxaemia, inflammatory cytokines, altered mineral metabolism and SCFA imbalance	Subchondral bone, osteoblasts, osteoclasts, infrapatellar fat pad and cartilage	May contribute to osteophyte formation, bone marrow lesion-like changes, osteochondral remodelling and metabolic OA phenotypes.	(35, 36, 77, 95–100, 118, 119)
Sex, ageing and postmenopausal modifiers	Oestrogen deficiency, high-fat-diet-induced dysbiosis and age-related microbial changes	Systemic immune-metabolic networks, synovium, bone, infrapatellar fat pad and cartilage	May modify gut microbiota-mediated inflammation, barrier dysfunction, immunosenescence, inflammaging and tissue-specific OA phenotypes.	(24, 42, 43, 59, 92–94, 150, 169)

AhR, aryl hydrocarbon receptor; HIF-2α, hypoxia-inducible factor-2α; IgA, immunoglobulin A; IL, interleukin; ILC3, group 3 innate lymphoid cell; LPS, lipopolysaccharide; MAPK, mitogen-activated protein kinase; MMP, matrix metalloproteinase; NF-κB, nuclear factor-κB; NLRP3, NOD-, LRR- and pyrin domain-containing protein 3; OA, osteoarthritis; RANKL, receptor activator of nuclear factor-κB ligand; SCFAs, short-chain fatty acids; Treg, regulatory T cell; TNF-α, tumour necrosis factor-α.

## 4 Microbiome-derived biomarkers for diagnosis and stratification

Early diagnosis and clinically meaningful stratification remain major challenges in OA. Conventional diagnosis relies on symptoms, physical examination and imaging evidence of structural joint damage. Pain and radiographic change, however, are often discordant, and structural abnormalities may become visible only after substantial tissue remodelling has occurred. Biomarkers are needed to identify risk, define phenotypes and monitor response. The gut microbiota is a potential source of non-invasive biomarkers. Microbial taxa, microbial genes and gut-derived metabolites in stool, blood or synovial fluid may reflect systemic inflammation, barrier dysfunction and metabolic perturbation. At present, these biomarkers should be considered complementary tools for risk assessment and stratification, not stand-alone diagnostic tests.

### 4.1 Taxonomic biomarkers

Taxonomic profiling of the gut microbiota has provided the most direct biomarker evidence in OA. Advances in 16S rRNA gene sequencing, shotgun metagenomic sequencing, metatranscriptomics and metabolomics have enabled increasingly detailed characterisation of microbial diversity, microbial function and host–microbe metabolic interactions in OA cohorts (40). Several studies have reported differences in gut microbial composition between patients with OA and healthy controls, including altered alpha and beta diversity, enrichment of potentially pro-inflammatory taxa and depletion of bacteria associated with anti-inflammatory metabolite production (37, 38, 54).

In symptomatic hand OA, increased abundance of genera such as Bilophila and Desulfovibrio, together with reduced Roseburia, has been linked to disease manifestations and perturbations in amino acid, carbohydrate and lipid metabolic pathways (37). These taxa are biologically plausible candidates because Desulfovibrio and Bilophila have been associated with inflammatory states, whereas Roseburia is a major butyrate-producing genus that may support intestinal barrier function and immune tolerance. In knee OA, other microbial signatures have been proposed, including altered abundance of Faecalibacterium, Prevotella, Anaerostipes hadrus and Blautia (54). Importantly, some metagenomic analyses have extended beyond bacteria and identified changes in the gut mycobiome and virome, suggesting that OA-associated dysbiosis may involve a broader multikingdom microbial disturbance rather than bacterial taxa alone (54).

Microbial taxa may also provide information about disease severity, symptoms and inflammatory state. Specific gut microbial profiles have been associated with clinical features, inflammatory markers and functional outcomes, suggesting potential utility for monitoring disease activity or identifying patients with inflammatory or metabolic OA phenotypes (13). In overweight individuals, a random forest model based on seven microbial genera, including Gemmiger, Klebsiella, Akkermansia and Prevotella, achieved an area under the curve of 83.36% for identifying individuals at risk of OA (39). This finding supports the possibility that microbial combinations, rather than single taxa, may improve risk prediction.

Causal-inference approaches have further identified candidate taxa associated with OA susceptibility. Mendelian randomisation analyses have suggested possible causal relationships between knee OA and microbial groups such as Methanobacteriaceae and Desulfovibrionales (55, 101). However, these results should be interpreted carefully. Taxonomic biomarkers remain vulnerable to limited resolution, population differences and host or environmental confounding. Therefore, although taxonomic biomarkers are promising, no single microbial signature is yet sufficiently validated for routine OA diagnosis. Future work should prioritise reproducibility across independent cohorts, standardised sample processing and integration with clinical phenotyping.

### 4.2 Metabolite biomarkers

Microbial metabolites may provide more functionally informative biomarkers than taxonomic composition alone. Whereas microbial taxa can vary widely between individuals, convergent metabolic outputs may more directly reflect biological mechanisms relevant to OA. Gut-derived metabolites can be measured in stool, serum, urine or synovial fluid and may capture intestinal microbial activity, host–microbe co-metabolism, systemic inflammation and local joint tissue responses.

LPS and related markers of microbial translocation are among the most mechanistically relevant candidates. Increased intestinal permeability can allow LPS and other microbial products to enter the circulation, where they activate Toll-like receptor signalling and promote low-grade systemic inflammation (40, 62–64). Serum lipopolysaccharide-binding protein, zonulin and related permeability markers may therefore serve as indirect indicators of gut barrier dysfunction and endotoxaemia. In OA studies, changes in LPS-related markers have been linked to inflammatory activation and clinical outcomes, supporting their potential value in identifying patients with a gut barrier–inflammatory phenotype (102).

SCFAs, including acetate, propionate and butyrate, are another major class of candidate biomarkers. These metabolites are produced by bacterial fermentation of dietary fibre and regulate epithelial barrier integrity, immune-cell differentiation, macrophage polarisation and chondrocyte homeostasis (50, 51, 66). Reduced SCFA-producing capacity may indicate loss of protective microbial functions, whereas restoration of SCFA levels may reflect response to diet, prebiotic or probiotic interventions. Because SCFAs can influence both intestinal immunity and cartilage or subchondral bone biology, their measurement may help link microbial function with OA-relevant tissue responses.

Synovial fluid metabolomics has identified gut microbiota-associated metabolites that may distinguish OA from other inflammatory arthropathies or reflect local joint pathology. Studies have detected elevated hippuric acid and indoleacetic acid in OA synovial fluid. Because both metabolites relate to microbial metabolism, these findings support the presence of gut-derived or host–microbe co-metabolites in the joint microenvironment (27). Serum metabolomic studies have also identified lipid mediators, including leukotriene B4 and prostaglandin D2, that correlate with osteophyte formation severity in knee OA (28). These metabolites may not be exclusively microbial in origin, but their association with inflammatory and structural features suggests that combined host–microbial metabolic signatures could improve phenotypic classification.

Tryptophan-derived metabolites are particularly attractive biomarkers because they also have direct mechanistic relevance. Gut bacteria convert tryptophan into indole derivatives that can activate aryl hydrocarbon receptor signalling and modulate chondrocyte inflammation (17, 67, 103, 104). Alterations in tryptophan metabolism have been associated with symptomatic and erosive hand OA, suggesting that these metabolites may help identify inflammatory or erosive phenotypes (103, 104). Bile acids represent another promising class. Microbiota-dependent bile acid transformation may influence macrophage polarisation, metabolic inflammation and synovial responses, and altered bile acid profiles have been implicated in OA-related mechanisms (16, 70).

Despite this promise, metabolite biomarkers face several challenges. Many metabolites are influenced by diet, circadian rhythm, liver function, renal clearance, medication use and comorbid metabolic disease. Moreover, distinguishing microbial origin from host metabolism is often difficult without paired metagenomic and metabolomic data. Thus, metabolite biomarkers should be interpreted as part of integrated biological networks rather than isolated diagnostic molecules.

### 4.3 Multi-omics and AI-assisted models

The most promising diagnostic strategy is likely to involve integration of microbiome data with metabolomic, transcriptomic, proteomic, imaging and clinical variables. OA is biologically heterogeneous, and a single microbial taxon or metabolite is unlikely to capture the complexity of disease initiation, symptom generation and structural progression. Multi-omics approaches can identify coordinated networks linking gut microbial composition, microbial metabolites and host inflammatory or catabolic responses.

Integrative studies have begun to reveal such networks. Analyses combining microbiome, metabolomic and host molecular data have identified neutrophil extracellular trap-associated genes, lipid metabolites and microbial taxa that correlate with OA severity and inflammation (105). These data suggest that microbiome-derived biomarkers may be most informative when embedded within host-response pathways involving lipid metabolism, immune activation and extracellular matrix remodelling. However, most reported microbe–host associations remain exploratory and require mechanistic validation before they can be interpreted as causal pathways.

Machine learning offers a framework for handling these high-dimensional datasets. Models that combine gut microbial profiles with clinical variables, inflammatory markers, pain scores and imaging findings have shown improved discrimination between OA patients and controls compared with individual biomarkers alone (29, 30, 39, 106). MRI-based assessment of synovitis, osteophytes and structural damage can be integrated with microbiome and metabolomic features to identify inflammatory or metabolic OA phenotypes (29). Similarly, serum metabolite profiles combined with microbial taxa, such as relationships between Bacteroides plebeius, Faecalibacterium prausnitzii, pyrogallol and 3-hydroxybutyrate, may capture systemic metabolic patterns linked to OA progression (31).

However, AI-assisted biomarker discovery must be approached cautiously. Models trained on small, single-centre datasets are prone to overfitting and may perform poorly in external populations. Batch effects, sequencing depth, taxonomic database selection, diet and medication exposure can all produce spurious signals. For clinical translation, microbiome-based models will need independent validation, transparent reporting, calibration across populations and demonstration that they improve decision-making beyond established clinical and imaging measures. At present, the most realistic clinical role for microbiome-derived biomarkers is not to diagnose OA independently, but to support stratification. Such biomarkers could help identify patients with gut barrier dysfunction, metabolic inflammation, low SCFA-producing capacity, bile acid dysregulation or inflammatory microbial signatures. These categories may eventually guide targeted interventions, including dietary fibre supplementation, probiotics, prebiotics, weight-loss strategies, microbial metabolite therapy or other microbiota-directed approaches.

In summary, microbiome-derived biomarkers offer a promising route towards more precise OA classification. Taxonomic signatures provide accessible but variable indicators of dysbiosis; metabolite biomarkers offer closer links to biological mechanisms; and multi-omics models may integrate microbial and host data into clinically meaningful endotypes. Before these approaches can enter routine practice, they must be validated in large longitudinal cohorts, standardised across analytical platforms and tested for their ability to predict progression, treatment response and clinically meaningful outcomes.

## 5 Therapeutic modulation of the microbiome

Recognition of the gut-joint axis has increased interest in microbiota-targeted interventions for OA. Unlike conventional treatments, which mainly address pain, inflammation or mechanical dysfunction after joint damage has occurred, microbiome-directed approaches aim to modify upstream systemic drivers such as barrier dysfunction, endotoxaemia, metabolic inflammation and immune imbalance. Candidate strategies include antibiotics, probiotics, prebiotics, synbiotics, dietary modulation, FMT, microbial metabolites and natural compounds that reshape gut microbial ecology. Therapeutic evidence remains uneven. Antibiotic and FMT studies are most useful for mechanism, probiotics and prebiotics are currently the most feasible clinical tools, and dietary approaches have broad translational potential because they target both microbial ecology and metabolic risk. Therefore, clinicians should consider microbiome modulation a promising adjunctive strategy, not an established disease-modifying therapy for OA.

### 5.1 Antibiotics: mechanistic tool rather than clinical solution

Antibiotic intervention has been useful for demonstrating that gut microbial communities can influence OA progression. In experimental models, broad-spectrum antibiotics can deplete or reshape intestinal microbiota, reduce circulating microbial products and alter systemic inflammatory tone. In destabilization of the medial meniscus models, antibiotics such as ampicillin and neomycin reduced serum LPS, TNF-α and IL-6 levels, decreased MMP-13 expression and attenuated cartilage degradation and osteophyte formation (45). Similar protective effects have been observed in ovariectomized models with combined OA and bone loss, in which antibiotic-induced microbiome depletion reduced LPS, inflammatory cytokines and cartilage injury while improving bone microarchitecture (77). These findings support the concept that gut-derived inflammatory signals can amplify osteochondral degeneration.

Antibiotics have also helped define temporal windows in which the microbiota may influence post-traumatic OA. In murine models, antibiotic administration before joint injury reduced inflammatory gene expression and promoted an anti-inflammatory macrophage phenotype, thereby slowing cartilage degeneration (107). Such studies suggest that the gut microbiota may influence early inflammatory responses after joint trauma, a critical window for disease initiation. However, the same findings also illustrate why antibiotics are better understood as experimental tools than as routine clinical interventions. Broad-spectrum antibiotic treatment removes both harmful and beneficial microbes, disrupts metabolite production, and may produce effects that are difficult to attribute to specific microbial taxa or pathways.

Safety concerns further limit the clinical use of antibiotics for OA. Long-term or repeated antibiotic exposure can reduce microbial diversity, promote opportunistic pathogens, impair intestinal barrier function and increase the burden of antibiotic resistance genes. In patients with periprosthetic joint infection, antibiotic therapy has been associated with marked gut dysbiosis, reduced microbial diversity and enrichment of opportunistic pathogens such as Escherichia and Klebsiella (108). Studies have also begun to examine antibiotic resistance genes and virulence factor genes in OA-associated gut microbiomes, highlighting potential risks of microbiome disruption (109). Moreover, some antibiotics may have extraintestinal effects unrelated to microbiota modulation, including neurobehavioral changes reported with ciprofloxacin in OA animal models (110). Therefore, although antibiotic studies support the biological relevance of the gut microbiota in OA, antibiotics themselves are unlikely to become a broadly acceptable microbiome-targeted therapy for chronic OA. Their main value lies in identifying microbial mechanisms, defining causal windows and informing safer, more selective approaches.

### 5.2 Probiotics, prebiotics and synbiotics

Probiotics represent one of the most actively studied microbiota-targeted interventions in OA. By introducing live microorganisms with potential health benefits, probiotics may restore microbial balance, strengthen intestinal barrier integrity, increase anti-inflammatory metabolites and modulate immune responses. In preclinical OA models, several probiotic strains or mixtures have shown anti-inflammatory and chondroprotective effects. For example, probiotics containing Lactobacillus and Bifidobacterium species reduced IL-1β, IL-6 and TNF-α in serum or synovial fluid, improved cartilage morphology and decreased OA histological scores (47, 48). Some effects appear to involve downregulation of TLR2, TLR4 and NF-κB signalling, suppression of MMP expression and restoration of anabolic–catabolic balance in cartilage (47, 48).

Specific strains may act through distinct mechanisms. Streptococcus thermophilus and Lactobacillus pentosus have been linked to increased γ-aminobutyric acid production, which may support cartilage anabolism and reduce catabolic responses (49). Latilactobacillus sakei LB-P12 attenuated OA progression by suppressing NF-κB and HIF-2α signalling and reducing MMP-13 expression (76). Lactobacillus kefiranofaciens has been reported to improve intestinal integrity, increase short-chain fatty acid (SCFA) levels, reduce oxidative stress and regulate matrix metalloproteinases in OA models (111). These findings emphasise that probiotic effects are strain-specific and cannot be generalised across all products labelled as probiotics.

Clinical evidence is encouraging but remains limited. Probiotic supplementation, including Saccharomyces boulardii and multi-strain formulations containing Lactobacillus and Bifidobacterium, has been associated with reductions in pain, inflammatory biomarkers such as high-sensitivity C-reactive protein, oxidative stress markers and improvements in physical function or quality of life in patients with OA (33, 112, 113). Probiotics may also enhance conventional nutraceutical therapy. Preliminary evidence from a single clinical study suggests that Probio-M8 combined with chondroitin sulphate may modulate gut microbial composition and improve clinical responses in knee OA; however, this finding requires confirmation in larger independent trials (114). Preclinical studies similarly suggest that probiotics combined with chondroitin sulphate can more effectively suppress TLR/NF-κB signalling and inflammatory cytokine production than either intervention alone (48).

Prebiotics offer a complementary strategy by selectively promoting beneficial microbial functions rather than introducing exogenous organisms. Non-digestible fibres such as inulin and oligofructose can increase SCFA-producing bacteria, improve epithelial barrier integrity and reduce systemic inflammation. In adults with obesity and knee OA, prebiotic supplementation has been associated with improved physical function, reduced trunk fat mass and changes in serum metabolomic pathways, including phenylalanine and tyrosine metabolism (115). Inulin supplementation and exercise have both been reported to reduce knee OA pain, potentially through increased butyrate production and modulation of glucagon-like peptide-1-related pathways (116). Experimental studies also suggest that prebiotics may restore tight junction proteins such as ZO-1 and occludin, thereby reducing gut permeability and downstream inflammatory activation (117).

Synbiotics, which combine probiotics and prebiotics, may offer advantages by improving colonisation, substrate availability and metabolite production. However, optimal combinations, dosage, treatment duration and patient selection remain undefined. Importantly, probiotic and prebiotic interventions are unlikely to benefit all OA patients equally. Their effects may depend on baseline microbiota composition, diet, obesity status, sex, metabolic phenotype and medication use. Future trials should therefore move beyond one-size-fits-all supplementation and adopt microbiome-informed designs that test strain-specific effects in biologically defined OA subgroups.

### 5.3 Diet, weight loss and metabolic reprogramming

Dietary modulation may be the most clinically feasible and safest route for targeting the gut microbiota in OA. Diet is a major determinant of microbial composition and metabolic output, and dietary interventions can simultaneously influence body weight, systemic inflammation, lipid metabolism, insulin resistance and intestinal barrier function. These effects are particularly relevant to metabolic OA, in which obesity and metabolic syndrome interact with gut dysbiosis and low-grade inflammation (118, 119).

High-fibre diets are especially relevant because dietary fibre is fermented by gut bacteria into SCFAs, including acetate, propionate and butyrate. These metabolites support epithelial barrier integrity, promote immune tolerance and may directly regulate chondrocyte and subchondral bone homeostasis (120, 121). In experimental OA, increased fibre intake has been associated with enrichment of beneficial bacterial taxa, reduced systemic inflammation and protection of joint tissues. One study linked high dietary fibre intake to increased Sestrin2 expression in knee joints, maintenance of chondrocyte activity and attenuation of synovial inflammation (65, 122). These findings suggest that fibre-mediated microbiome modulation may reduce the systemic inflammatory burden that contributes to OA progression.

Anti-inflammatory dietary patterns may also benefit OA through microbiome-dependent and microbiome-independent mechanisms. Omega-3 polyunsaturated fatty acids, polyphenols and Mediterranean-style dietary patterns can reduce oxidative stress, lipid-mediated inflammation and cartilage catabolic signalling (123). Although direct clinical evidence linking these diets, microbiome changes and OA structural outcomes remains limited, their broader metabolic benefits make them attractive adjuncts, especially for patients with obesity-associated OA. Dietary approaches that reduce saturated fat and refined sugar while increasing fibre, plant-derived polyphenols and fermented foods may partially restore microbial balance and reduce inflammatory mediators in preclinical models (124, 125).

Natural products and traditional dietary compounds provide additional examples of microbiome-modulating therapies. Liubao tea and pterostilbene have been reported to alter gut microbiota, reduce TNF-α and IL-6 expression and alleviate OA progression in experimental models (75, 126). Mulberry water extract alleviated OA symptoms by enriching beneficial bacteria such as Lactobacillus johnsonii, restoring bile acid metabolism and regulating macrophage polarisation (70). Isopsoralen improved OA-related inflammation and bone abnormalities by modulating gut microbiota composition, SCFA levels and MAPK/NF-κB signalling (127). Gut microbiota-derived metabolites such as urolithin A and urolithin B have also been shown to reduce chondrocyte ferroptosis and inflammation through AMPK/mTOR/HIF-1α and FGFR3/NCOA4/FTH1-related pathways (71, 72). These findings are mechanistically intriguing, but most remain preclinical and require pharmacokinetic, safety and efficacy validation.

Clinicians should include weight loss in microbiome-targeted OA management because it affects both mechanical load and metabolic inflammation. Reductions in adiposity can improve insulin sensitivity, reduce adipokine-driven inflammation and reshape gut microbial communities. Combining weight management with high-fibre diets, prebiotics, probiotics and exercise may therefore produce synergistic effects on the gut–metabolism–joint axis. However, clinical trials must distinguish the effects of microbiome modulation from the benefits of weight reduction itself.

### 5.4 FMT and next-generation microbial therapeutics

FMT provides the most direct method for restructuring the gut microbiota. By transferring a complex microbial community from a screened donor to a recipient, FMT aims to restore microbial diversity and function. In OA, preclinical FMT studies support the concept that microbial communities can influence disease severity. In mice with antibiotic-depleted microbiota, FMT combined with probiotic administration inhibited DMM-induced cartilage damage and improved subchondral bone parameters (52). In germ-free mice, transplantation of microbiota from OA donors produced different effects depending on donor obesity status, demonstrating that microbial communities can transmit disease-modifying inflammatory and metabolic traits (53). FMT has also shown benefit in rat OA models by improving gait abnormalities, reducing inflammation and mitigating cartilage destruction (128).

Despite these promising data, FMT remains far from routine OA therapy. Clinical evidence is still sparse, and FMT carries substantial safety, ethical and regulatory challenges. Donor microbiota composition varies widely, preparation and administration protocols are difficult to standardise, and there is risk of pathogen transmission, immune reactions and transfer of undesirable metabolic or inflammatory traits (12, 129, 130). These risks are especially important in a chronic, non-life-threatening disease such as OA, where the acceptable safety threshold is high. Regulatory frameworks for FMT also differ across jurisdictions, with some classifying it as a biological product, drug or tissue-based therapy (131). Thus, FMT may be most useful as a research tool for causal testing and for identifying therapeutic microbial consortia, rather than as a near-term clinical intervention for OA.

Next-generation microbial therapeutics may overcome some limitations of FMT. These approaches include defined bacterial consortia, engineered probiotics, postbiotics, purified microbial metabolites and receptor-targeted compounds. Compared with FMT, defined products offer better standardisation, quality control, dosing precision and safety monitoring. SCFA-based therapies, bile acid derivatives, tryptophan metabolites and urolithins represent examples of metabolite-focussed strategies that may reproduce beneficial microbial functions without transferring whole microbial communities (132–134). Engineered microbes could theoretically be designed to produce anti-inflammatory metabolites, reinforce barrier integrity or deliver therapeutic molecules locally in the gut. However, such strategies remain experimental and will require careful evaluation of ecological stability, off-target effects, long-term colonisation and regulatory oversight.

In summary, therapeutic modulation of the microbiome offers a promising but still evolving strategy for OA. Antibiotic studies have clarified causal mechanisms but are limited by safety and non-specificity; probiotics, prebiotics and synbiotics are clinically feasible but require strain-specific and phenotype-specific validation; diet and weight management provide low-risk approaches with broad metabolic benefits; and FMT or next-generation microbial therapeutics may eventually enable more precise manipulation of the gut–joint axis. The key challenge is to move from broad microbiome modulation to mechanism-based intervention: identifying which patients have microbiome-driven OA endotypes, which microbial pathways are therapeutically actionable, and which interventions produce durable improvements in pain, function and structural progression.

## 6 From experimental promise to clinical translation

Microbiome research has generated a compelling framework for OA: intestinal dysbiosis may contribute to joint degeneration through barrier dysfunction, microbial metabolites, systemic inflammation and immune-metabolic remodelling. Translation from experimental promise to clinical application remains incomplete. The field has moved from observational profiling to interventional studies using prebiotics, probiotics, synbiotics, dietary modulation and microbial metabolites, but these approaches have not reached standardised, guideline-supported OA care. Future studies must address three barriers before clinical translation is realistic: methodological standardisation, rigorous trial design, and robust safety, regulatory and ethical frameworks.

### 6.1 Standardisation of microbiome studies

A central obstacle in translating gut microbiota research into OA practice is the lack of methodological standardisation. Current studies vary widely in stool sampling procedures, storage conditions, DNA extraction protocols, sequencing platforms, bioinformatic pipelines, taxonomic databases and statistical methods. These technical differences can substantially alter microbial profiles and limit comparability across cohorts. Although 16S rRNA sequencing remains widely used because of its relatively low cost and high throughput, it provides limited species- or strain-level resolution and offers little direct information about microbial function (135, 136). Shotgun metagenomic sequencing provides deeper taxonomic and functional information, including microbial genes and metabolic pathways, but it is more expensive, computationally demanding and less accessible for routine clinical use (56, 137).

Standardised sample handling is particularly important. Stool samples should be collected using protocols that minimise contamination and preserve microbial DNA and metabolites, ideally through immediate freezing or validated stabilisation buffers. DNA extraction methods should be harmonised because different protocols can preferentially lyse certain bacterial taxa and introduce systematic bias. Bioinformatic workflows, including quality control, sequence denoising, taxonomic assignment, diversity analysis and batch-effect correction, must also be reported transparently. Commonly used diversity indices, such as Shannon diversity and Bray–Curtis dissimilarity, provide useful summary measures but are insufficient for clinical interpretation without standardised reference ranges and disease-relevant thresholds (60, 138–140).

Another challenge is biological variability. Gut microbiota composition differs substantially between individuals and is influenced by age, sex, diet, geography, body mass index, medication exposure, comorbidities, physical activity and recent antibiotic use (141–143). OA itself is heterogeneous, encompassing post-traumatic, metabolic, inflammatory, ageing-associated and postmenopausal phenotypes. Therefore, microbiome studies that treat OA as a single disease category are unlikely to produce reproducible biomarkers. Future studies should collect detailed metadata, including diet, analgesic and anti-inflammatory drug use, antibiotic exposure, metabolic status and OA phenotype. Harmonised reporting standards would allow investigators to distinguish disease-associated microbial signals from background variation (11, 144, 145).

Multi-omics integration may help overcome some limitations of taxonomic profiling. Combining 16S rRNA sequencing or metagenomics with metabolomics, proteomics, transcriptomics and host inflammatory markers can identify functional microbial pathways more closely linked to OA biology (146). For example, microbial group-enhanced models have been proposed to predict knee OA progression, and faecal metabolomics has been used to characterise OA-associated metabolic alterations (41, 119). However, multi-omics approaches also increase analytical complexity and require large sample sizes, external validation and transparent modelling (147, 148). For clinical translation, microbiome-derived tools must be reproducible, interpretable and demonstrably superior to existing clinical and imaging-based assessments.

### 6.2 Clinical trial design

Clinical trials of microbiota-targeted interventions in OA are still at an early stage. Existing studies include observational cohorts, Mendelian randomisation analyses and randomised controlled trials testing probiotics, prebiotics, synbiotics, diet-related interventions and adjunctive therapies (149–151). Some trials have shown encouraging results. Oligofructose-enriched inulin improved physical function, reduced fat mass and altered gut microbial composition in adults with obesity and knee OA (116). Bifidobacterium animalis subsp. lactis Probio-M8 used with chondroitin sulphate alleviated symptoms and modulated immune and microbial features in postmenopausal women with knee OA (152–154). Electroacupuncture has also been associated with changes in gut microbial composition that correlated with symptom improvement in knee OA patients (155). These studies support the therapeutic potential of the gut–joint axis but are not yet sufficient to establish microbiota-based interventions as disease-modifying treatments.

Future clinical trials should focus on clearly defined OA endotypes rather than broad diagnostic categories. Patients with obesity-associated OA, inflammatory synovitis, postmenopausal OA, low short-chain fatty acid-producing capacity or elevated markers of gut permeability may respond differently to microbiota-targeted therapies. Trialists should incorporate baseline microbiome and metabolomic profiling into study design to stratify participants and identify responders. Trials should also predefine whether the intervention is intended to reduce pain, improve function, modify inflammation, alter structural progression or prevent disease onset in high-risk populations.

Endpoint selection is another key issue. Pain scales and WOMAC scores are clinically meaningful but are sensitive to placebo effects, expectation, analgesic use and physical activity. Structural endpoints, including radiographic joint-space narrowing, MRI-detected cartilage loss, bone marrow lesions, synovitis and osteophyte progression, require longer follow-up but provide stronger evidence for disease modification. Biomarkers such as high-sensitivity C-reactive protein, LPS-binding protein, zonulin, inflammatory cytokines, cartilage degradation markers and microbial metabolites may help bridge symptomatic and structural outcomes (156–158). Ideally, future trials should combine patient-reported outcomes, objective functional assessments, imaging biomarkers and microbiome-related mechanistic endpoints.

Trial duration is especially important. OA is a chronic disease, and short-term symptom improvement does not necessarily indicate sustained biological modification of the joint. Microbiota-targeted interventions may require weeks to months to reshape microbial ecosystems and even longer to influence structural outcomes. Longitudinal sampling before, during and after treatment is needed to determine whether microbial changes are durable, whether they precede clinical improvement and whether loss of microbial response predicts symptom recurrence. Such designs would help distinguish causal therapeutic mechanisms from transient or incidental microbiome fluctuations.

### 6.3 Safety, regulation and ethics

Safety is central to clinical translation because OA usually requires long-term management, and many patients are older adults with comorbidities. Probiotics and prebiotics are generally considered low risk, and several clinical studies have reported favourable tolerability (159, 160). For example, multi-strain probiotic formulations have been associated with reduced walking-related pain and lower high-sensitivity C-reactive protein levels in OA patients (151). However, safety cannot be assumed across all products, strains, doses or patient populations. Probiotic effects are strain-specific, and commercial preparations differ in viability, purity, dose, manufacturing quality and contaminant risk. Immunocompromised patients, frail older adults and patients with altered gut barrier function may require particular caution.

Postbiotics and microbial metabolites may offer more standardised alternatives to live microorganisms. Non-viable microbial products are theoretically more stable and may reduce risks associated with colonisation or infection. However, clinical efficacy remains uncertain; for example, postbiotic preparations have shown acceptable safety but have not consistently demonstrated superiority over placebo in OA trials (161). Similarly, microbial metabolite-based therapies, bile acid modulators and engineered microbial products require careful pharmacokinetic, toxicological and ecological evaluation before clinical use. Their systemic effects may extend beyond joints to immune, metabolic and neurological pathways.

FMT poses the greatest translational and ethical challenge. Although animal studies support its ability to modify OA-related phenotypes, clinical application in OA is premature. FMT involves transfer of complex microbial communities, with potential risks of pathogen transmission, immune reactions, metabolic trait transfer and long-term ecological disruption (131). Donor screening, manufacturing standards, route of administration, dose, repeat treatment schedules and recipient monitoring remain difficult to standardise. Because OA is chronic and rarely immediately life-threatening, the acceptable risk threshold for FMT is much lower than in severe recurrent intestinal infections. At present, FMT should be regarded primarily as a research tool for causal testing and microbial discovery rather than a routine OA therapy.

Regulatory frameworks for microbiome-based interventions remain fragmented. Depending on the jurisdiction and product type, probiotics, synbiotics, FMT, postbiotics and engineered microbial products may be regulated as foods, supplements, biologics, drugs or tissue-derived therapies (162). This inconsistency complicates trial design, manufacturing oversight, quality control and clinical implementation. Clear regulatory standards are needed for product characterisation, potency assays, contamination testing, adverse event reporting and long-term follow-up. Ethical review should also address informed consent, especially when interventions may produce durable changes in microbial ecology or uncertain systemic effects.

Ultimately, clinical translation will require multidisciplinary collaboration among orthopaedic surgeons, rheumatologists, microbiologists, immunologists, bioinformaticians, nutrition scientists, regulatory experts and trialists. Such collaboration is essential for designing studies that are biologically grounded, clinically relevant and methodologically rigorous. The goal should not be to add microbiome testing indiscriminately to OA care, but to identify specific situations in which microbiome information improves diagnosis, stratification, therapeutic selection or monitoring. Until then, gut microbiota-targeted strategies should be presented as promising adjuncts supported by growing mechanistic evidence, but not yet as established replacements for evidence-based OA management. Several unresolved controversies and knowledge gaps that currently limit interpretation and clinical translation in this field are summarized in **Box 1**.

Box 1 **Outstanding controversies and knowledge gaps in gut microbiota–OA research**

1. Authenticity of synovial microbial signals

Synovial fluid is a low-biomass sample; contamination from reagents, skin, collection tubes and sequencing pipelines must be excluded using negative controls, mock communities and independent validation.

2. Conflicting evidence for endotoxaemia in human OA

Animal models support LPS-driven inflammation, but human data are inconsistent. The study by Loeser et al. suggested that increased serum LPS may be linked to obesity-related OA, whereas consistent microbial dysbiosis was not detected.

3. Causality versus consequence

Dysbiosis may precede OA, result from pain-related inactivity and dietary changes, or reflect obesity, medication and comorbidities.

4. Inconsistent probiotic efficacy

Clinical effects vary by strain, dose, duration, baseline microbiota, OA phenotype, BMI, diet and outcome definition. Therefore, probiotics should not be presented as broadly effective.

5. FMT and ecological risk

FMT may transfer beneficial and harmful traits. Long-term engraftment, ecological instability, antimicrobial resistance genes and jurisdiction-specific regulation remain unresolved.

## 7 Precision medicine and future directions

The long-term value of gut microbiota research in OA will depend on whether it moves beyond descriptive association and contributes to precision medicine. OA is clinically and biologically heterogeneous. Patients differ by joint site, structural severity, pain phenotype, metabolic status, inflammatory burden, sex, age, hormonal state, diet and medication exposure. A single microbial taxon, metabolite or intervention is therefore unlikely to apply to all patients. Instead, the gut microbiota should be considered one component of a broader host-microbe-metabolism network that may help define biologically meaningful OA endotypes and guide individualised prevention or treatment.

### 7.1 Defining gut-driven OA endotypes

A key future direction is the identification of gut-driven OA endotypes. Current OA classification is still largely based on symptoms, affected joint site and imaging findings, but these features do not fully capture underlying biological heterogeneity. Microbiome-based stratification may help distinguish patients in whom gut barrier dysfunction, microbial metabolite imbalance or systemic low-grade inflammation contributes substantially to disease progression. For example, patients with elevated markers of intestinal permeability, reduced short-chain fatty acid-producing capacity, altered bile acid metabolism or pro-inflammatory microbial signatures may represent a “gut-inflamed” OA endotype (11, 163, 164).

Metabolic OA is one of the most plausible contexts in which microbiome-informed endotyping could be clinically useful. Obesity and metabolic syndrome are associated with gut dysbiosis, endotoxaemia, adipose tissue inflammation, insulin resistance and altered circulating metabolites, all of which can influence synovitis, cartilage catabolism and subchondral bone remodelling (62, 118, 119). In such patients, microbiota-targeted interventions may be most effective when combined with weight reduction, dietary fibre enrichment, exercise and metabolic risk control. Conversely, mechanically driven post-traumatic OA may require different therapeutic priorities, with microbiome modulation serving as an adjunct only in patients with evidence of systemic inflammatory activation.

Future endotyping should integrate microbial taxonomy with microbial function. Taxonomic signatures alone are often unstable across cohorts, whereas functional pathways and metabolites may provide more reproducible biological signals. Multi-omics approaches combining metagenomics, metabolomics, transcriptomics, proteomics and inflammatory biomarkers can identify networks linking microbial species, microbial metabolites and host catabolic pathways (31, 105, 145). Such integrated profiles may eventually classify patients according to dominant mechanisms, such as barrier dysfunction, endotoxin-driven inflammation, SCFA deficiency, bile acid dysregulation, macrophage polarisation or ferroptosis-related cartilage injury (70–72, 75).

Machine learning and artificial intelligence may accelerate this process by analysing complex microbiome and clinical datasets. Predictive models incorporating microbial genera, serum metabolites, imaging features and clinical parameters have already shown potential for OA risk identification and disease stratification (66, 165, 166). However, these models must be externally validated and biologically interpretable. The goal should not be to generate black-box classifiers, but to identify actionable endotypes that inform treatment choice, prognosis and monitoring. Digital twin approaches, which integrate longitudinal clinical, imaging, molecular and behavioural data, may eventually allow simulation of individualised treatment responses in musculoskeletal disease (167, 168). For OA, such approaches remain aspirational but conceptually aligned with the complexity of the gut–joint axis.

### 7.2 Sex, ageing and metabolic status as biological modifiers

Precision microbiome medicine in OA must account for sex, ageing and metabolic status. These variables shape both the gut microbiota and OA biology, and they may explain part of the heterogeneity in current studies. Sex differences are particularly important because women have a higher prevalence of OA after midlife, and postmenopausal hormonal changes influence inflammation, bone remodelling and intestinal microbial ecology. Estrogen deficiency has been associated with gut dysbiosis, increased intestinal permeability and systemic low-grade inflammation, which may contribute to both skeletal and joint degeneration (42). Experimental studies also show that high-fat diet-induced changes in gut microbial diversity, metabolic dysfunction and joint pathology differ between males and females (43, 169).

These findings imply that future microbiome studies should treat sex as a biological variable rather than a confounder to be adjusted away. Trials of probiotics, prebiotics, diet or microbial metabolites should be powered to detect sex-specific effects, especially in postmenopausal women, who may have distinct interactions among estrogen deficiency, microbial dysbiosis, bone remodelling and inflammatory OA pathways (42, 43). Sex-specific microbial or metabolite signatures could also help identify patients who are more likely to benefit from targeted interventions.

Ageing is another major modifier. Age-related changes in gut microbiota composition, immune senescence, reduced barrier integrity and chronic low-grade inflammation may interact with cartilage senescence and impaired tissue repair (24, 170, 171). Older patients may have reduced microbial resilience and may respond differently to dietary or probiotic interventions than younger patients. They may also be more susceptible to adverse effects from aggressive microbiome manipulation, particularly FMT or live microbial products (172, 173). Future studies should therefore include age-stratified analyses and long-term safety monitoring.

Metabolic status may be the strongest practical variable for patient selection. Obesity-associated knee OA is characterised by interactions between mechanical load, systemic metabolic inflammation and gut dysbiosis (43, 119). Clinical studies suggest that prebiotic supplementation, probiotic therapy and combined interventions may improve pain, physical function and inflammatory markers in overweight or obese OA populations (113, 115). These findings support a future strategy in which patients are stratified by body composition, insulin resistance, lipid profile, inflammatory biomarkers and gut microbial function before microbiota-targeted treatment is selected.

### 7.3 From association to intervention

The next phase of research must shift from association to intervention. Many studies have identified differences in microbial taxa or metabolites between OA patients and controls, but fewer have shown that modifying those microbial features changes disease trajectory. Establishing causality will require longitudinal human cohorts, repeated microbiome and metabolomic sampling, intervention trials, mechanistic validation in animal models and causal-inference approaches such as Mendelian randomisation (174, 175). Importantly, microbial changes should be linked to clinically meaningful endpoints, including pain, function, imaging progression, inflammatory biomarkers and structural tissue outcomes.

Clinical trials should adopt adaptive and mechanism-based designs. Instead of enrolling unselected OA populations, future studies should focus on biologically defined subgroups, such as patients with obesity-associated OA, elevated gut permeability markers, reduced SCFA levels, inflammatory synovitis or postmenopausal metabolic inflammation. Interventions should be chosen according to the presumed mechanism: fibre or prebiotics for low SCFA-producing capacity, strain-specific probiotics for barrier and immune modulation, dietary interventions for metabolic dysbiosis, and microbial metabolites for defined signalling pathways (176–178). This approach would help determine whether microbiota-targeted therapies are most effective in patients with specific gut–joint axis abnormalities.

Another priority is the development of standardised and clinically usable microbiome tests. For precision medicine, biomarkers must be reproducible, affordable, interpretable and actionable. A stool sequencing result is unlikely to be useful unless it leads to a clear clinical decision. Therefore, future biomarker panels may need to combine microbial taxa, microbial genes, metabolite levels and host inflammatory markers into risk scores or treatment-response classifiers (179, 180). These panels should be validated across diverse populations and compared against standard clinical models.

Combination therapy will probably be more effective than microbiome modulation alone. OA involves mechanical, inflammatory, metabolic and psychosocial components, and gut microbiota-targeted approaches should be integrated with exercise, weight management, analgesia, rehabilitation, nutritional counselling and, when appropriate, conventional pharmacological or procedural therapies (116, 155, 181). The aim is not to replace established OA care, but to add a biologically informed layer that addresses systemic drivers of disease. Such combined strategies may be particularly relevant for patients with obesity, metabolic syndrome or persistent inflammatory symptoms despite standard management. Finally, future research must maintain a realistic balance between innovation and clinical caution. The gut microbiota is a promising therapeutic target, but it is also a complex ecosystem with wide inter-individual variability and potential long-term consequences of manipulation. Large randomised controlled trials, standardised endpoints, transparent reporting and long-term safety assessment are essential before microbiome-based interventions can be recommended as routine OA care. The field should therefore progress from the broad concept of “modulating the microbiome” towards precise, mechanism-based and patient-specific interventions.

## 8 Conclusion

The gut microbiota is an emerging but not yet clinically established contributor to osteoarthritis. Current evidence suggests that intestinal dysbiosis may influence OA through an immune-metabolic gut–joint axis, involving barrier dysfunction, microbial metabolite imbalance, microbial product translocation and reprogramming of mucosal, systemic and joint immunity. Rather than acting through a single pathway, dysbiosis may promote OA by reshaping macrophage polarisation, inflammasome activity, Th17/Treg balance, mucosal IgA responses, innate lymphoid cell and γδT-cell function, and age-related inflammaging. These immune changes can converge on synovitis, chondrocyte catabolism, extracellular matrix degradation, subchondral bone remodelling and pain-related inflammatory signalling.

Despite growing mechanistic support from animal models, germ-free systems and faecal microbiota transplantation studies, human causality remains incompletely defined. Major uncertainties include OA phenotype heterogeneity, confounding by obesity, diet, medication and physical activity, the authenticity of low-biomass microbial signals in synovial fluid, inconsistent evidence for endotoxaemia in human OA and variable responses to probiotic or microbiota-directed interventions. Therefore, microbiome-derived biomarkers and microbiota-targeted therapies should currently be regarded as promising adjunctive strategies rather than validated diagnostic tools or disease-modifying treatments. Future studies should move beyond descriptive microbial profiling towards longitudinal, multi-omics and mechanism-based research integrating standardised microbiome analysis, contamination controls, immune phenotyping and rigorously designed clinical trials. Such work will be essential for determining whether gut–joint axis biology can be translated into microbiome-informed precision care for OA.

## References

[1] Xi Y Wang Z Wei Y Xiao N Duan L Zhao T . Gut microbiota and osteoarthritis: From pathogenesis to novel therapeutic opportunities. Am J Chin Med. (2025) 53:43–66. doi: 10.1142/s0192415x2550003x 39880660

[2] Petersen KK Ciampi de Andrade D Pickering G Giordano R Hertel E Edwards RR . The complexity of pain in osteoarthritis. Nat Rev Rheumatol. (2026) 22:378–89. doi: 10.1038/s41584-026-01372-8 42062510

[3] Fan Y Pedersen O . Gut microbiota in human metabolic health and disease. Nat Rev Microbiol. (2021) 19:55–71. doi: 10.1038/s41579-020-0433-9 32887946

[4] Zheng D Liwinski T Elinav E . Interaction between microbiota and immunity in health and disease. Cell Res. (2020) 30:492–506. doi: 10.1038/s41422-020-0332-7 32433595 PMC7264227

[5] Iliev ID Ananthakrishnan AN Guo CJ . Microbiota in inflammatory bowel disease: mechanisms of disease and therapeutic opportunities. Nat Rev Microbiol. (2025) 23:509–24. doi: 10.1038/s41579-025-01163-0 40065181 PMC12289240

[6] Iatcu CO Steen A Covasa M . Gut microbiota and complications of type-2 diabetes. Nutrients. (2021) 14:166. doi: 10.3390/nu14010166 35011044 PMC8747253

[7] Ma F Li Z Liu H Chen S Zheng S Zhu J . Dietary-timing-induced gut microbiota diurnal oscillations modulate inflammatory rhythms in rheumatoid arthritis. Cell Metab. (2024) 36:2367–82:e5. doi: 10.1016/j.cmet.2025.05.007 39260371

[8] Zheng XQ Wang DB Jiang YR Song CL . Gut microbiota and microbial metabolites for osteoporosis. Gut Microbes. (2025) 17:2437247. doi: 10.1080/19490976.2024.2437247 39690861 PMC11657146

[9] Claesson MJ Jeffery IB Conde S Power SE O'Connor EM Cusack S . Gut microbiota composition correlates with diet and health in the elderly. Nature. (2012) 488:178–84. doi: 10.1038/nature11319 22797518

[10] Jeyaraman M Ram PR Jeyaraman N Yadav S . The gut-joint axis in osteoarthritis. Cureus. (2023) 15:e48951. doi: 10.7759/cureus.48951 38106807 PMC10725653

[11] Sun C Zhou X Guo T Meng J . The immune role of the intestinal microbiome in knee osteoarthritis: a review of the possible mechanisms and therapies. Front Immunol. (2023) 14:1168818. doi: 10.3389/fimmu.2023.1168818 37388748 PMC10306395

[12] Zhang YW Li RY Wu Y Wang P Zhou QR Su JC . Gut microbiota and bone aging: Focusing on the gut-X axis modes. J Orthop Translat. (2026) 57:101064. doi: 10.1016/j.jot.2026.101064 41777702 PMC12950467

[13] Hao X Shang X Liu J Chi R Zhang J Xu T . The gut microbiota in osteoarthritis: where do we stand and what can we do? Arthritis Res Ther. (2021) 23:42. doi: 10.1186/s13075-021-02427-9 33504365 PMC7839300

[14] Boer CG Radjabzadeh D Medina-Gomez C Garmaeva S Schiphof D Arp P . Intestinal microbiome composition and its relation to joint pain and inflammation. Nat Commun. (2019) 10:4881. doi: 10.1038/s41467-019-12873-4 31653850 PMC6814863

[15] Mukhopadhya I Louis P . Gut microbiota-derived short-chain fatty acids and their role in human health and disease. Nat Rev Microbiol. (2025) 23:635–51. doi: 10.1038/s41579-025-01183-w 40360779

[16] Li J Ye J Yang T Hunter DJ Zhang W Doherty M . Bile acids metabolism in symptomatic hand osteoarthritis. Arthritis Rheumatol. (2025). doi: 10.1002/art.70048 41451559

[17] Zhuang H Ren X Jiang F Zhou P . Indole-3-propionic acid alleviates chondrocytes inflammation and osteoarthritis via the AhR/NF-kappaB axis. Mol Med. (2023) 29:17. doi: 10.1186/s10020-023-00614-9 36721094 PMC9890697

[18] Fu L Zhang P Wang Y Liu X . Microbiota-bone axis in ageing-related bone diseases. Front Endocrinol (Lausanne). (2024) 15:1414350. doi: 10.3389/fendo.2024.1414350 39076510 PMC11284018

[19] Pandiyan P Bhaskaran N Zou M Schneider E Jayaraman S Huehn J . Microbiome dependent regulation of T(regs) and Th17 cells in mucosa. Front Immunol. (2019) 10:426. doi: 10.3389/fimmu.2019.00426 30906299 PMC6419713

[20] Jia Y He T Wu D Tong J Zhu J Li Z . The treatment of Qibai Pingfei Capsule on chronic obstructive pulmonary disease may be mediated by Th17/Treg balance and gut-lung axis microbiota. J Transl Med. (2022) 20:281. doi: 10.1186/s12967-022-03481-w 35729584 PMC9210581

[21] Yang W Yu T Huang X Bilotta AJ Xu L Lu Y . Intestinal microbiota-derived short-chain fatty acids regulation of immune cell IL-22 production and gut immunity. Nat Commun. (2020) 11:4457. doi: 10.1038/s41467-020-18262-6 32901017 PMC7478978

[22] Dupraz L Magniez A Rolhion N Richard ML Da Costa G Touch S . Gut microbiota-derived short-chain fatty acids regulate IL-17 production by mouse and human intestinal gammadelta T cells. Cell Rep. (2021) 36:109332. doi: 10.1016/j.celrep.2021.109332 34233192

[23] Conrey PE Denu L O'Boyle KC Rozich I Green J Maslanka J . IgA deficiency destabilizes homeostasis toward intestinal microbes and increases systemic immune dysregulation. Sci Immunol. (2023) 8:eade2335. doi: 10.1126/sciimmunol.ade2335 37235682 PMC11623094

[24] Carrasco E Gomez de Las Heras MM Gabande-Rodriguez E Desdin-Mico G Aranda JF Mittelbrunn M . The role of T cells in age-related diseases. Nat Rev Immunol. (2022) 22:97–111. doi: 10.1038/s41577-021-00557-4 34099898

[25] Guo Q Jin Y Chen X Ye X Shen X Lin M . NF-kappaB in biology and targeted therapy: new insights and translational implications. Signal Transduct Target Ther. (2024) 9:53. doi: 10.1038/s41392-024-01757-9 38433280 PMC10910037

[26] Jiang L Liu S Kong H . Microbiome-augmented model for predicting knee osteoarthritis progression based on gut microbiota and Kellgren-Lawrence classification. Cureus. (2024) 16:e73402. doi: 10.7759/cureus.73402 39664130 PMC11631569

[27] Kaplan O Karalilova R Batalov Z Batalov K Kazakova M Sarafian V . Synovial joint fluid metabolomic profiles and pathways differentiate osteoarthritis, rheumatoid arthritis, and psoriatic arthritis. Metabolites. (2026) 16:70. doi: 10.21203/rs.3.rs-7526932/v1 41590678 PMC12844152

[28] Zhu D Wang X Xi Z Chen K Feng Y Zi C . Diet influences knee osteoarthritis osteophyte formation via gut microbiota and serum metabolites. iScience. (2024) 27:110111. doi: 10.1016/j.isci.2024.110111 38957790 PMC11217616

[29] Wang X Liu Y Sun Z Li J Lu Z Huang J . Multi-omics reveal the dysregulated gut-joint axis in knee synovitis: Data from two osteoarthritis studies in China. Adv Sci (Weinh). (2026) 13:e12020. doi: 10.1002/advs.202512020 41354462 PMC12866873

[30] Kurz C Arbeeva L Azcarate-Peril MA Stewart DA Lascelles BDX Loeser RF . Exploring associations among pro-inflammatory cytokines, osteoarthritis, and gut microbiome composition in individuals with obesity using machine learning. Osteoarthr Cartil Open. (2025) 7:100603. doi: 10.1016/j.ocarto.2025.100603 40213471 PMC11985103

[31] Wang W Liu X Nan H Li H Yan L . Specific gut microbiota and serum metabolite changes in patients with osteoarthritis. Front Cell Dev Biol. (2025) 13:1543510. doi: 10.3389/fcell.2025.1543510 40027098 PMC11868077

[32] Yu F Zhu C Wu W . Senile osteoarthritis regulated by the gut microbiota: From mechanisms to treatments. Int J Mol Sci. (2025) 26:1505. doi: 10.3390/ijms26041505 40003971 PMC11855920

[33] Karim A Khan HA Ahmad F Qaisar R . Probiotics improve functional performance in patients with osteoarthritis: a randomized placebo-controlled clinical trial. Eur J Nutr. (2025) 64:290. doi: 10.1007/s00394-025-03805-8 41065805

[34] Karim A . Unveiling the potential of probiotics in osteoarthritis management. Curr Rheumatol Rep. (2024) 27:2. doi: 10.1007/s11926-024-01166-5 39579259

[35] Li H Wang J Hao L Huang G . Exploring the interconnection between metabolic dysfunction and gut microbiome dysbiosis in osteoarthritis: a narrative review. Biomedicines. (2024) 12:2182. doi: 10.3390/biomedicines12102182 39457494 PMC11505131

[36] Huang J Liu M Zhang H Sun G Furey A Rahman P . Altered gut microbiota and host pathways in obesity-related knee osteoarthritis. Clin Exp Rheumatol. (2026). doi: 10.55563/clinexprheumatol/ksopba 41562358

[37] Wei J Zhang C Zhang Y Zhang W Doherty M Yang T . Association between gut microbiota and symptomatic hand osteoarthritis: Data from the Xiangya Osteoarthritis Study. Arthritis Rheumatol. (2021) 73:1656–62. doi: 10.1002/art.41729 33760399 PMC8457181

[38] Liu S Li G Zhu Y Xu C Yang Q Xiong A . Analysis of gut microbiome composition, function, and phenotype in patients with osteoarthritis. Front Microbiol. (2022) 13:980591. doi: 10.3389/fmicb.2022.980591 36504782 PMC9732244

[39] Wang Z Zhu H Jiang Q Zhu YZ . The gut microbiome as non-invasive biomarkers for identifying overweight people at risk for osteoarthritis. Microb Pathog. (2021) 157:104976. doi: 10.1016/j.micpath.2021.104976 34023440

[40] Aydin M Avci GA Yilmaz UI Avci E . A new approach to osteoarthritis: gut microbiota. Rev Assoc Med Bras (1992). (2025) 71:e20241528. doi: 10.1590/1806-9282.20241528 40332266 PMC12051967

[41] Gilat R Yazdi AA Weissman AC Joyce KM Bouftas FA Muth SA . The gut microbiome and joint microbiome show alterations in patients with knee osteoarthritis versus controls: a systematic review. Arthroscopy. (2025) 41:1226–38. doi: 10.1016/j.arthro.2024.05.010 38797504

[42] Kverka M Stepan JJ . Associations among estrogens, the gut microbiome and osteoporosis. Curr Osteoporos Rep. (2024) 23:2. doi: 10.1007/s11914-024-00896-w 39585466 PMC11588883

[43] Griffin TM Lopes EBP Cortassa D Batushansky A Jeffries MA Makosa D . Sexually dimorphic metabolic effects of a high fat diet on knee osteoarthritis in mice. Biol Sex Differ. (2024) 15:103. doi: 10.1186/s13293-024-00680-6 39639386 PMC11619521

[44] Yan Y Yi X Duan Y Jiang B Huang T Inglis BM . Alteration of the gut microbiota in rhesus monkey with spontaneous osteoarthritis. BMC Microbiol. (2021) 21:328. doi: 10.1186/s12866-021-02390-0 34837955 PMC8627091

[45] Guan Z Jia J Zhang C Sun T Zhang W Yuan W . Gut microbiome dysbiosis alleviates the progression of osteoarthritis in mice. Clin Sci (Lond). (2020) 134:3159–74. doi: 10.1042/cs20201224 33215637

[46] Liu S Xu H Liu L Ma W Fan H Liu F . Gut microbiome dysbiosis accelerates osteoarthritis progression by inducing IFP-SM inflammation in "double-hit" mice. Arthritis Res Ther. (2025) 27:137. doi: 10.1186/s13075-025-03602-y 40624668 PMC12232632

[47] Korotkyi O Dvorshchenko K Falalyeyeva T Sulaieva O Kobyliak N Abenavoli L . Combined effects of probiotic and chondroprotector during osteoarthritis in rats. Panminerva Med. (2020) 62:93–101. doi: 10.23736/s0031-0808.20.03841-0 32192320

[48] Korotkyi O Huet A Dvorshchenko K Kobyliak N Falalyeyeva T Ostapchenko L . Probiotic composition and chondroitin sulfate regulate TLR-2/4-mediated NF-kappaB inflammatory pathway and cartilage metabolism in experimental osteoarthritis. Probiotics Antimicrob Proteins. (2021) 13:1018–32. doi: 10.1007/s12602-020-09735-7 33459997

[49] Amin U Jiang R Raza SM Fan M Liang L Feng N . Gut-joint axis: Oral probiotic ameliorates osteoarthritis. J Tradit Complement Med. (2024) 14:26–39. doi: 10.1016/j.jtcme.2023.06.002 38223812 PMC10785157

[50] Korsten S Hartog M Berends AJ Koenders MI Popa CD Vromans H . A sustained-release butyrate tablet suppresses ex vivo T helper cell activation of osteoarthritis patients in a double-blind placebo-controlled randomized trial. Nutrients. (2024) 16:3384. doi: 10.3390/nu16193384 39408351 PMC11478393

[51] Han S Cho KH Na HS Jhun J Moon YM Choi JW . Propionate attenuates osteoarthritis progression by regulating the gut-joint axis. Front Immunol. (2026) 17:1717556. doi: 10.3389/fimmu.2026.1717556 41890750 PMC13012969

[52] Sophocleous A Azfer A Huesa C Stylianou E Ralston SH . Probiotics inhibit cartilage damage and progression of osteoarthritis in mice. Calcif Tissue Int. (2023) 112:66–73. doi: 10.1007/s00223-022-01030-7 36261653 PMC9813193

[53] Ran C Xu Y Wang Q Cao H Li D Wang Y . Gut microbiota from osteoarthritic patients without obesity aggravates osteoarthritis progression in rats by enriching acetic acid. Microb Pathog. (2025) 207:107911. doi: 10.1016/j.micpath.2025.107911 40683544

[54] Chen C Zhang Y Yao X Li S Wang G Huang Y . Characterizations of the gut bacteriome, mycobiome, and virome in patients with osteoarthritis. Microbiol Spectr. (2023) 11:e0171122. doi: 10.1128/spectrum.01711-22 36515546 PMC9927108

[55] Yu XH Yang YQ Cao RR Bo L Lei SF . The causal role of gut microbiota in development of osteoarthritis. Osteoarthritis Cartilage. (2021) 29:1741–50. doi: 10.1016/j.joca.2021.08.003 34425228

[56] Di J Xi Y Wu Y Di Y Xing X Zhang Z . Gut microbiota metabolic pathways: Key players in knee osteoarthritis development. Exp Gerontol. (2024) 196:112566. doi: 10.1016/j.exger.2024.112566 39226947

[57] Lee YH Song GG . The gut microbiome and osteoarthritis: A two-sample mendelian randomization study. J Rheum Dis. (2021) 28:94–100. doi: 10.4078/jrd.2021.28.2.94 37476017 PMC10324885

[58] Gleason B Chisari E Parvizi J . Osteoarthritis can also start in the gut: The gut-joint axis. Indian J Orthop. (2022) 56:1150–5. doi: 10.1007/s43465-021-00473-8 35813544 PMC9232669

[59] Abughazaleh N Smith H Seerattan RA Hart DA Reimer RA Herzog W . Development of shoulder osteoarthritis and bone lesions in female and male rats subjected to a high fat/sucrose diet. Sci Rep. (2024) 14:25871. doi: 10.1038/s41598-024-76703-4 39468197 PMC11519393

[60] Balouei F Rivera C Paradis A Stefanon B Kelly S McCarthy N . Gut microbiota variation in aging dogs with osteoarthritis. Anim (Basel). (2025) 15:1619. doi: 10.20944/preprints202504.1427.v1 PMC12153520 40509085

[61] Stevens C Norris S Arbeeva L Carter S Enomoto M Nelson AE . Gut microbiome and osteoarthritis: Insights from the naturally occurring canine model of osteoarthritis. Arthritis Rheumatol. (2024) 76:1758–63. doi: 10.1002/art.42956 39030898 PMC11605265

[62] Loeser RF Arbeeva L Kelley K Fodor AA Sun S Ulici V . Association of increased serum lipopolysaccharide, but not microbial dysbiosis, with obesity-related osteoarthritis. Arthritis Rheumatol. (2022) 74:227–36. doi: 10.1126/sageke.2004.35.dn2 34423918 PMC8795472

[63] Guido G Ausenda G Iascone V Chisari E . Gut permeability and osteoarthritis, towards a mechanistic understanding of the pathogenesis: a systematic review. Ann Med. (2021) 53:2380–90. doi: 10.1080/07853890.2021.2014557 34933614 PMC8725942

[64] Li K Liu A Zong W Dai L Liu Y Luo R . Moderate exercise ameliorates osteoarthritis by reducing lipopolysaccharides from gut microbiota in mice. Saudi J Biol Sci. (2021) 28:40–9. doi: 10.1016/j.sjbs.2020.08.027 33424281 PMC7783636

[65] Wu Y Li X Meng H Wang Y Sheng P Dong Y . Dietary fiber may benefit chondrocyte activity maintenance. Front Cell Infect Microbiol. (2024) 14:1401963. doi: 10.3389/fcimb.2024.1401963 38803575 PMC11129558

[66] Han J Meng X Kong H Li X Chen P Zhang XA . Links between short-chain fatty acids and osteoarthritis from pathology to clinic via gut-joint axis. Stem Cell Res Ther. (2025) 16:251. doi: 10.1186/s13287-025-04386-3 40390010 PMC12090658

[67] Zhuang H Li B Xie T Xu C Ren X Jiang F . Indole-3-aldehyde alleviates chondrocytes inflammation through the AhR-NF-kappaB signalling pathway. Int Immunopharmacol. (2022) 113:109314. doi: 10.1016/j.intimp.2022.109314 36252481

[68] Binvignat M Emond P Mifsud F Miao B Courties A Lefevre A . Serum tryptophan metabolites are associated with erosive hand osteoarthritis and pain: results from the DIGICOD cohort. Osteoarthritis Cartilage. (2023) 31:1132–43. doi: 10.1016/j.joca.2023.04.007 37105396

[69] Chen L Huang Z Li Q Chen C Luo Y Kang P . Activated intestinal microbiome-associated tryptophan metabolism upregulates aryl hydrocarbon receptor to promote osteoarthritis in a rat model. Int Immunopharmacol. (2023) 118:110020. doi: 10.1016/j.intimp.2023.110020 36933489

[70] Zhao JA Zheng YZ Wang F Ye JM Guo YF Liang XD . Mulberry water extract alleviates osteoarthritis via Lactobacillus johnsonii-dependent bile acid restoration. Phytomedicine. (2026) 150:157679. doi: 10.1016/j.phymed.2025.157679 41421286

[71] Wang X Xu Q Huang J Gao Y Zheng F Liu G . Urolithin A protects mice against osteoarthritis by inhibiting chondrocyte ferroptosis through activating AMPK/mTOR/HIF-1alpha signaling pathway. J Nutr Biochem. (2026) 147:110111. doi: 10.1016/j.jnutbio.2025.110111 40945544

[72] Li Y Gu H Jiang X Li J Li Q Song K . Gut microbiota metabolite urolithin B inhibits chondrocyte ferroptosis by rewriting iron homeostasis via FGFR3/NCOA4/FTH1 axis, alleviating osteoarthritis. Phytomedicine. (2025) 148:157292. doi: 10.1016/j.phymed.2025.157292 41005058

[73] Guan Z Jin X Guan Z Liu S Tao K Luo L . The gut microbiota metabolite capsiate regulate SLC2A1 expression by targeting HIF-1alpha to inhibit knee osteoarthritis-induced ferroptosis. Aging Cell. (2023) 22:e13807. doi: 10.1111/acel.13807 36890785 PMC10265160

[74] Chang YT Huang KC Pranata R Chen YL Chen SN Cheng YH . Evaluation of the protective effects of chondroitin sulfate oligosaccharide against osteoarthritis via inactivation of NLRP3 inflammasome by *in vivo* and *in vitro* studies. Int Immunopharmacol. (2024) 142:113148. doi: 10.1016/j.intimp.2024.113148 39276449

[75] Lee YC Chang YT Cheng YH Pranata R Hsu HH Chen YL . Pterostilbene protects against osteoarthritis through NLRP3 inflammasome inactivation and improves gut microbiota as evidenced by *in vivo* and *in vitro* studies. J Agric Food Chem. (2024) 72:9150–63. doi: 10.1021/acs.jafc.3c09749 38624135 PMC11046483

[76] Song M Kim WJ Shim J Song K . Latilactobacillus sakei LB-P12 ameliorates osteoarthritis by reducing cartilage degradation and inflammation via regulation of NF-kappaB/HIF-2alpha pathway. J Microbiol Biotechnol. (2025) 35:e2504013. doi: 10.4014/jmb.2504.04013 40329628 PMC12089955

[77] Yin Q Gu J Ren P Guan Z Wang Y Bai R . Microbiome dysbiosis by antibiotics protects cartilage degradation in OAOP mice. J Endocrinol. (2024) 261:e230330. doi: 10.1530/joe-23-0330 38265817

[78] Wei J Zhang Y Xie C Lei G Zeng C . The gut-joint axis in osteoarthritis. Nat Rev Rheumatol. (2026) 22:345–61. doi: 10.1038/s41584-026-01378-2 42098429

[79] Perez-Lopez A Behnsen J Nuccio SP Raffatellu M . Mucosal immunity to pathogenic intestinal bacteria. Nat Rev Immunol. (2016) 16:135–48. doi: 10.1038/nri.2015.17 26898110

[80] Di Vincenzo F Del Gaudio A Petito V Lopetuso LR Scaldaferri F . Gut microbiota, intestinal permeability, and systemic inflammation: a narrative review. Intern Emerg Med. (2024) 19:275–93. doi: 10.1007/s11739-023-03374-w 37505311 PMC10954893

[81] Yuan Z Jiang D Yang M Tao J Hu X Yang X . Emerging roles of macrophage polarization in osteoarthritis: Mechanisms and therapeutic strategies. Orthop Surg. (2024) 16:532–50. doi: 10.1111/os.13993 38296798 PMC10925521

[82] Zhang Y Ji Q . Macrophage polarization in osteoarthritis progression: a promising therapeutic target. Front Cell Dev Biol. (2023) 11:1269724. doi: 10.3389/fcell.2023.1269724 37954210 PMC10639142

[83] Li YS Luo W Zhu SA Lei GH . T cells in osteoarthritis: Alterations and beyond. Front Immunol. (2017) 8:356. doi: 10.3389/fimmu.2017.00356 28424692 PMC5371609

[84] de Lange-Brokaar BJ Ioan-Facsinay A van Osch GJ Zuurmond AM Schoones J Toes RE . Synovial inflammation, immune cells and their cytokines in osteoarthritis: a review. Osteoarthritis Cartilage. (2012) 20:1484–99. doi: 10.1016/j.joca.2012.08.027 22960092

[85] Ohara D Takeuchi Y Hirota K . Type 17 immunity: novel insights into intestinal homeostasis and autoimmune pathogenesis driven by gut-primed T cells. Cell Mol Immunol. (2024) 21:1183–200. doi: 10.1038/s41423-024-01218-x 39379604 PMC11528014

[86] McBride DA Dorn NC Yao M Johnson WT Wang W Bottini N . Short-chain fatty acid-mediated epigenetic modulation of inflammatory T cells *in vitro*. Drug Delivery Transl Res. (2023) 13:1912–24. doi: 10.1007/s13346-022-01284-6 36566262 PMC10695156

[87] Lee JG Lee J Lee AR Jo SV Park CH Han DS . Impact of short-chain fatty acid supplementation on gut inflammation and microbiota composition in a murine colitis model. J Nutr Biochem. (2022) 101:108926. doi: 10.1016/j.jnutbio.2021.108926 34848335

[88] Burt KG Scanzello CR . B cells in osteoarthritis: simply a sign or a target for therapy? Osteoarthritis Cartilage. (2023) 31:1148–51. doi: 10.1016/j.joca.2023.06.002 37328048 PMC10680778

[89] Lu P Li Y Yang S Yao H Tu B Ning R . B cell activation, differentiation, and their potential molecular mechanisms in osteoarthritic synovial tissue. J Inflammation Res. (2025) 18:2137–51. doi: 10.2147/jir.s503597 39959649 PMC11829641

[90] Wang H Wang T He Z Wen C Huang L Wang M . Deciphering the role of innate lymphoid cells group 3 in the gut microenvironment: A narrative review of their novel contributions to autoimmune disease pathogenesis. J Inflammation Res. (2025) 18:5741–57. doi: 10.2147/jir.s512652 40322535 PMC12048713

[91] Mohamed AA Al-Ramadi BK Fernandez-Cabezudo MJ . Interplay between microbiota and gammadelta T cells: Insights into immune homeostasis and neuro-immune interactions. Int J Mol Sci. (2024) 25:1747. doi: 10.3390/ijms25031747 38339023 PMC10855551

[92] Thomas R Wang W Su DM . Contributions of age-related thymic involution to immunosenescence and inflammaging. Immun Ageing. (2020) 17:2. doi: 10.1186/s12979-020-0173-8 31988649 PMC6971920

[93] Goyani P Christodoulou R Vassiliou E . Immunosenescence: Aging and immune system decline. Vaccines (Basel). (2024) 12:1314. doi: 10.3390/vaccines12121314 39771976 PMC11680340

[94] Liu Y Duan J Dang Y Hao R Wang H Tan E . Remodeling of senescent macrophages in synovium alleviates trauma- and aging-induced osteoarthritis. Bioact Mater. (2026) 55:42–56. doi: 10.1016/j.bioactmat.2025.09.016 41035432 PMC12481617

[95] Hansdah K Lui JC . Emerging insights into the endocrine regulation of bone homeostasis by gut microbiome. J Endocr Soc. (2024) 8:bvae117. doi: 10.1210/jendso/bvae117 38957653 PMC11215793

[96] Shi H Huang L Zhang JH Shen C Zhang N Lv C . Gut microbiota regulates brain-bone axis to influence osteoporosis pathogenesis and treatment. Res (Wash D C). (2026) 9:1178. doi: 10.34133/research.1178 41847216 PMC12989651

[97] Ozaki D Kubota R Maeno T Abdelhakim M Hitosugi N . Association between gut microbiota, bone metabolism, and fracture risk in postmenopausal Japanese women. Osteoporos Int. (2021) 32:145–56. doi: 10.1007/s00198-020-05728-y 33241467 PMC7755620

[98] Zhang YW Cao MM Li YJ Lu PP Dai GC Zhang M . Fecal microbiota transplantation ameliorates bone loss in mice with ovariectomy-induced osteoporosis via modulating gut microbiota and metabolic function. J Orthop Translat. (2022) 37:46–60. doi: 10.1016/j.jot.2022.08.003 36196151 PMC9520092

[99] Mirmohammadali SN Gallant KMH Biruete A . Oh, my gut! New insights on the role of the gastrointestinal tract and the gut microbiome in chronic kidney disease-mineral and bone disorder. Curr Opin Nephrol Hypertens. (2024) 33:226–30. doi: 10.1097/mnh.0000000000000961 38088374 PMC11957419

[100] Lu L Tang M Li J Xie Y Li Y Xie J . Gut microbiota and serum metabolic signatures of high-fat-induced bone loss in mice. Front Cell Infect Microbiol. (2021) 11:788576. doi: 10.3389/fcimb.2021.788576 35004355 PMC8727351

[101] Wang X Wu Y Liu Y Chen F Chen S Zhang F . Altered gut microbiome profile in patients with knee osteoarthritis. Front Microbiol. (2023) 14:1153424. doi: 10.3389/fmicb.2023.1153424 37250055 PMC10213253

[102] Karim A Khan HA Ahmad F Qaisar R . Butyrate (short-chain fatty acid) alleviates lipopolysaccharide-binding proteins and improves physical function in knee osteoarthritis patients. Int J Biol Macromol. (2025) 307:142017. doi: 10.1016/j.ijbiomac.2025.142017 40081693

[103] Wei J Yang Z Li J Zhang Y Zhang W Doherty M . Association between gut microbiome-related metabolites and symptomatic hand osteoarthritis in two independent cohorts. EBioMedicine. (2023) 98:104892. doi: 10.1016/j.ebiom.2023.104892 38006743 PMC10775900

[104] Xie D Wang Y Li J Yang T Zhang Y Zhang W . Correlation between gut dysbiosis, metabolite alterations and erosive hand osteoarthritis - an observational study within the community-based Xiangya Osteoarthritis (XO) cohort. Osteoarthritis Cartilage. (2025) 33:1246–57. doi: 10.1016/j.joca.2025.07.004 40633803

[105] Xiang Y Li J Hu X Yang X Sun F Yang J . Combined multi-omics approach to identify the key metabolites, key microorganisms and biomarkers correlated with the neutrophil extracellular traps-associated gene TIMP1 in osteoarthritis. Front Pharmacol. (2025) 16:1665228. doi: 10.3389/fphar.2025.1665228 41170378 PMC12568690

[106] de Sire A Mancuso E Marotta N Massimino M Zito R Averta C . Association between gut microbiota composition and physical functioning in patients with knee osteoarthritis: a machine learning study. Sci Rep. (2025) 15:40826. doi: 10.1038/s41598-025-24500-y 41258193 PMC12630774

[107] Mendez ME Murugesh DK Sebastian A Hum NR McCloy SA Kuhn EA . Antibiotic treatment prior to injury improves post-traumatic osteoarthritis outcomes in mice. Int J Mol Sci. (2020) 21:6424. doi: 10.3390/ijms21176424 32899361 PMC7503363

[108] Muzhabaier K Li Y Wang F Guo X Chen Q Zhang X . Differential analysis of gut microbiome in patients with periprosthetic joint infection, aseptic failure, and osteoarthritis. Zhongguo Xiu Fu Chong Jian Wai Ke Za Zhi. (2026) 40:548–56. doi: 10.7507/1002-1892.202601002 PMC13096846 41981426

[109] Guo Y Feng H Du L Yu Z . Patterns of antibiotic resistance genes and virulence factor genes in the gut microbiome of patients with osteoarthritis and rheumatoid arthritis. Front Microbiol. (2024) 15:1427313. doi: 10.3389/fmicb.2024.1427313 39633808 PMC11615078

[110] Kekesi G Ducza E Gality H Buki A Toth K Tuboly G . Neurobehavioral impairments in ciprofloxacin-treated osteoarthritic adult rats. Ideggyogy Sz. (2023) 76:327–37. doi: 10.18071/isz.76.0327 37782061

[111] Jia L Yuan J Chen Y Liang P Wu J Xie Y . Lactobacillus kefiranofaciens and its enhancement effect on the anti-inflammatory function of CII/Gln in MIA-induced osteoarthritis by protecting the intestinal barrier and gut microecology. Int J Food Sci Nutr. (2025) 76:530–43. doi: 10.1080/09637486.2025.2508173 40401730

[112] Tian M Zhu Y Lu S Qin Y Li X Wang T . Clinical efficacy of probiotic supplementation in the treatment of knee osteoarthritis: a meta-analysis. Front Microbiol. (2025) 16:1526690. doi: 10.3389/fmicb.2025.1526690 40276226 PMC12020436

[113] Dolatkhah N Jafari A Eslamian F Toopchizadeh V Saleh P Hashemian M . Saccharomyces boulardii improves clinical and paraclinical indices in overweight/obese knee osteoarthritis patients: a randomized triple-blind placebo-controlled trial. Eur J Nutr. (2024) 63:2291–305. doi: 10.1007/s00394-024-03428-5 38761281

[114] Wang K Wang H Zhao Z Shen X Zhao J Zhang H . Bifidobacterium animalis subsp. lactis Probio-M8 enhances chondroitin efficacy for knee osteoarthritis in postmenopausal women via the gut-joint axis. mSystems. (2025) 10:e0086225. doi: 10.1128/msystems.00862-25 41313018 PMC12710372

[115] Fortuna R Wang W Mayengbam S Tuplin EWN Sampsell K Sharkey KA . Effect of prebiotic fiber on physical function and gut microbiota in adults, mostly women, with knee osteoarthritis and obesity: a randomized controlled trial. Eur J Nutr. (2024) 63:2149–61. doi: 10.1007/s00394-024-03415-w 38713231

[116] Kouraki A Franks S Vijay A Kurien T Taylor MA Smith SL . Effect of prebiotic supplementation with and without physiotherapy on pain and pain sensitivity in people with knee osteoarthritis. Nutrients. (2026) 18:714. doi: 10.3390/nu18050714 41829888 PMC12986947

[117] Mi Y Yi N Xu X Zeng F Li N Tan X . Prebiotics alleviate cartilage degradation and inflammation in post-traumatic osteoarthritic mice by modulating the gut barrier and fecal metabolomics. Food Funct. (2023) 14:4065–77. doi: 10.1039/d3fo00775h 37077156

[118] Jimenez-Muro M Soriano-Romani L Mora G Ricciardelli D Nieto JA . The microbiota-metabolic syndrome axis as a promoter of metabolic osteoarthritis. Life Sci. (2023) 329:121944. doi: 10.1016/j.lfs.2023.121944 37453577

[119] Rushing BR McRitchie S Arbeeva L Nelson AE Azcarate-Peril MA Li YY . Fecal metabolomics reveals products of dysregulated proteolysis and altered microbial metabolism in obesity-related osteoarthritis. Osteoarthritis Cartilage. (2022) 30:81–91. doi: 10.1016/j.joca.2021.10.006 34718137 PMC8712415

[120] Han X Ma Y Ding S Fang J Liu G . Regulation of dietary fiber on intestinal microorganisms and its effects on animal health. Anim Nutr. (2023) 14:356–69. doi: 10.1016/j.aninu.2023.06.004 37635930 PMC10448034

[121] Chen K Hu M Tang M Gao C Wang H Man S . Oligosaccharide and short-chain fatty acid: a double-edged sword in obese mice by regulating food intake and fat synthesis. Food Res Int. (2022) 159:111619. doi: 10.1016/j.foodres.2022.111619 35940810

[122] Kasprzyk N Nandy S Grygiel-Gorniak B . Diet in knee osteoarthritis-myths and facts. Nutrients. (2025) 17:1872. doi: 10.3390/nu17111872 40507141 PMC12157890

[123] Wang Y Cao Z Gao Y Shao P Gao S Dong M . Nutritional interventions for osteoarthritis: targeting the metabolism-inflammation-oxidative stress axis-clinical evidence and translational practice. Front Nutr. (2025) 12:1661136. doi: 10.3389/fnut.2025.1661136 41220710 PMC12597758

[124] Xiang W Ji B Jiang Y Xiang H . Association of low-grade inflammation caused by gut microbiota disturbances with osteoarthritis: a systematic review. Front Vet Sci. (2022) 9:938629. doi: 10.3389/fvets.2022.938629 36172610 PMC9510893

[125] Chisari E Wouthuyzen-Bakker M Friedrich AW Parvizi J . The relation between the gut microbiome and osteoarthritis: a systematic review of literature. PloS One. (2021) 16:e0261353. doi: 10.1371/journal.pone.0261353 34914764 PMC8675674

[126] Le G Wen R Huang Z Fang H Zheng J Wang Y . Integrating network pharmacology, microbiomics, and metabolomics to uncover the therapeutic effect of Liubao tea on osteoarthritis. Front Immunol. (2026) 17:1746350. doi: 10.3389/fimmu.2026.1746350 41798939 PMC12960094

[127] Zhang A Li S Qiao J Zhong C Zhang Z Ye X . Isopsoralen alleviates osteoarthritis by modulating the MAPK/NF-kappaB signaling pathway and regulating the structure of gut microbiota. J Ethnopharmacol. (2026) 361:121257. doi: 10.1016/j.jep.2026.121257 41628870

[128] Zheng YZ Chen QR Yang HM Zhao JA Ren LZ Wu YQ . Modulation of gut microbiota by crude mulberry polysaccharide attenuates knee osteoarthritis progression in rats. Int J Biol Macromol. (2024) 262:129936. doi: 10.1016/j.ijbiomac.2024.129936 38309391

[129] Huang Z Chen J Li B Zeng B Chou CH Zheng X . Faecal microbiota transplantation from metabolically compromised human donors accelerates osteoarthritis in mice. Ann Rheum Dis. (2020) 79:646–56. doi: 10.1136/annrheumdis-2019-216471 32205337 PMC7384301

[130] Cold F Svensson CK Petersen AM Hansen LH Helms M . Long-term safety following faecal microbiota transplantation as a treatment for recurrent Clostridioides difficile infection compared with patients treated with a fixed bacterial mixture: results from a retrospective cohort study. Cells. (2022) 11:435. doi: 10.3390/cells11030435 35159245 PMC8834574

[131] Jeyaraman N Jeyaraman M Dhanpal P Ramasubramanian S Ragavanandam L Muthu S . Gut microbiome and orthopaedic health: bridging the divide between digestion and bone integrity. World J Orthop. (2024) 15:1135–45. doi: 10.5312/wjo.v15.i12.1135 39744736 PMC11686522

[132] Korczak M Roszkowski P Skowronska W Zoldak KM Popowski D Granica S . Urolithin A conjugation with NSAIDs inhibits its glucuronidation and maintains improvement of Caco-2 monolayers' barrier function. BioMed Pharmacother. (2023) 169:115932. doi: 10.1016/j.biopha.2023.115932 38000358

[133] Ares AM Toribio L Garcia-Villalba R Villalgordo JM Althobaiti Y Tomas-Barberan FA . Separation of isomeric forms of urolithin glucuronides using supercritical fluid chromatography. J Agric Food Chem. (2023) 71:3033–9. doi: 10.1021/acs.jafc.2c07145 36719954 PMC9936581

[134] Zhao H Song G Zhu H Qian H Pan X Song X . Pharmacological effects of urolithin A and its role in muscle health and performance: current knowledge and prospects. Nutrients. (2023) 15:4441. doi: 10.3390/nu15204441 37892516 PMC10609777

[135] Curry KD Wang Q Nute MG Tyshaieva A Reeves E Soriano S . Emu: species-level microbial community profiling of full-length 16S rRNA Oxford Nanopore sequencing data. Nat Methods. (2022) 19:845–53. doi: 10.1038/s41592-022-01520-4 35773532 PMC9939874

[136] Callahan BJ Grinevich D Thakur S Balamotis MA Yehezkel TB . Ultra-accurate microbial amplicon sequencing with synthetic long reads. Microbiome. (2021) 9:130. doi: 10.1186/s40168-021-01072-3 34090540 PMC8179091

[137] Lewis S Nash A Li Q Ahn TH . Comparison of 16S and whole genome dog microbiomes using machine learning. Biodata Min. (2021) 14:41. doi: 10.1186/s13040-021-00270-x 34419136 PMC8379800

[138] Yang Y Li G Xie Y Wang L Lagler TM Yang Y . iSMNN: batch effect correction for single-cell RNA-seq data via iterative supervised mutual nearest neighbor refinement. Brief Bioinform. (2021) 22:bbab122. doi: 10.1093/bib/bbab122 33839756 PMC8579191

[139] Hui HWH Kong W Goh WWB . Thinking points for effective batch correction on biomedical data. Brief Bioinform. (2024) 25:bbae515. doi: 10.1093/bib/bbae515 39397427 PMC11471903

[140] Hu X Li H Chen M Qian J Jiang H . Reference-informed evaluation of batch correction for single-cell omics data with overcorrection awareness. Commun Biol. (2025) 8:521. doi: 10.1038/s42003-025-07947-7 40158033 PMC11954866

[141] Prochazkova N Falony G Dragsted LO Licht TR Raes J Roager HM . Advancing human gut microbiota research by considering gut transit time. Gut. (2023) 72:180–91. doi: 10.1136/gutjnl-2022-328166 PMC9763197 36171079

[142] Delaroque C Wu GD Compher C Ni J Albenberg L Liu Q . Diet standardization reduces intra-individual microbiome variation. Gut Microbes. (2022) 14:2149047. doi: 10.1080/19490976.2022.2149047 36426908 PMC9704386

[143] Favari C Rinaldi de Alvarenga JF Sanchez-Martinez L Tosi N Mignogna C Cremonini E . Factors driving the inter-individual variability in the metabolism and bioavailability of (poly)phenolic metabolites: a systematic review of human studies. Redox Biol. (2024) 71:103095. doi: 10.1016/j.redox.2024.103095 38428187 PMC10912651

[144] Bonato A Zenobi-Wong M Barreto G Huang Z . A systematic review of microbiome composition in osteoarthritis subjects. Osteoarthritis Cartilage. (2022) 30:786–801. doi: 10.1016/j.joca.2021.12.006 34958936

[145] Liu S Li G Xu H Wang Q Wei Y Yang Q . Cross-talk" between gut microbiome dysbiosis and osteoarthritis progression: a systematic review. Front Immunol. (2023) 14:1150572. doi: 10.3389/fimmu.2023.1150572 37180142 PMC10167637

[146] Shi C Cheng L Yu Y Chen S Dai Y Yang J . Multi-omics integration analysis: tools and applications in environmental toxicology. Environ pollut. (2024) 360:124675. doi: 10.1016/j.envpol.2024.124675 39103035

[147] Fukushima-Nomura A Kawasaki H Amagai M . Integrative omics redefining allergy mechanisms and precision medicine. Allergol Int. (2025) 74:514–24. doi: 10.1016/j.alit.2025.08.007 40975691

[148] Kimotho RN Maina S . Unraveling plant-microbe interactions: can integrated omics approaches offer concrete answers? J Exp Bot. (2024) 75:1289–313. doi: 10.1093/jxb/erad448 37950741 PMC10901211

[149] Li R Boer CG Oei L Medina-Gomez C . The gut microbiome: a new frontier in musculoskeletal research. Curr Osteoporos Rep. (2021) 19:347–57. doi: 10.1007/s11914-021-00675-x 33864574 PMC8310472

[150] Gaspar MG Nunez-Carro C Blanco-Blanco M Blanco FJ de Andres MC . Inflammaging contributes to osteoarthritis development and human microbiota variations and vice versa: a systematic review. Osteoarthritis Cartilage. (2025) 33:218–30. doi: 10.1016/j.joca.2024.11.005 39612977

[151] Moyseos M Michael J Ferreira N Sophocleous A . The effect of probiotics on the management of pain and inflammation in osteoarthritis: A systematic review and meta-analysis of clinical studies. Nutrients. (2024) 16:2243. doi: 10.3390/nu16142243 39064686 PMC11279588

[152] Monteiro SS Santos NC Almeida RLJ de Lima TLB Tome AES Morais SKQ . Evaluation of sapodilla pulp as a matrix for probiotic fermentation: Physicochemical changes, antioxidant potential, and *in vitro* digestibility during storage. Int J Food Microbiol. (2025) 435:111175. doi: 10.1016/j.ijfoodmicro.2025.111175 40139105

[153] Lin M Li S Lin J Wan J Xu T Qi X . The adjunct use of Bifidobacterium animalis subsp. lactis BAMA-B06/BAu-B0111 improves the therapeutic efficacy of Liuwei'anxiao Capsule in alleviating constipation in Parkinson's disease: A randomized controlled study. J Ethnopharmacol. (2026) 367:121694. doi: 10.1016/j.jep.2026.121694 41990927

[154] Jantama SS Pichayajittipong P Maneewong RY Cheng KC Jantama K . Transcriptional analysis of oxidative-tolerant and temperature-sensitive genes of Bifidobacterium animalis BF052 during freeze-drying process and development of its soymilk-synbiotic product containing banana and jicama powders. Food Res Int. (2025) 221:117600. doi: 10.1016/j.foodres.2025.117600 41185348

[155] Guo X Guo L Lu QZ Xie H Chen J Su WL . Effect of electroacupuncture combined with Tuina therapy on gut microbiota in patients with knee osteoarthritis. World J Gastroenterol. (2025) 31:105495. doi: 10.3748/wjg.v31.i18.105495 40496364 PMC12146932

[156] Ruan X Gu J Chen M Zhao F Aili M Zhang D . Multiple roles of ALK3 in osteoarthritis. Bone Joint Res. (2023) 12:397–411. doi: 10.1302/2046-3758.127.bjr-2022-0310.r1 37394235 PMC10315222

[157] Shi Y Shen S Huang D Yilamu K Chen Z Wang K . Delivery of FBXO6 with highly branched poly(beta-amino ester)s to modulate the inflammatory environment for the treatment of osteoarthritis. J Control Release. (2025) 378:294–305. doi: 10.1016/j.jconrel.2024.12.019 39674232

[158] Zhang Y Li J Liu J Gao Y Li K Zhao X . Ferroptosis in osteoarthritis: Towards novel therapeutic strategy. Cell Prolif. (2025) 58:e13779. doi: 10.1111/cpr.13779 39624950 PMC11882765

[159] Karim A Khan HA Shahid Iqbal M Ahmad F Qaisar R . The effect of multi-strain probiotics on frailty in osteoarthritis patients: A randomized trial focusing on intestinal leak repair. Eur J Clin Nutr. (2026) 80:506–14. doi: 10.1038/s41430-026-01719-0 41876860

[160] Zeng L Deng Y He Q Yang K Li J Xiang W . Safety and efficacy of probiotic supplementation in 8 types of inflammatory arthritis: A systematic review and meta-analysis of 34 randomized controlled trials. Front Immunol. (2022) 13:961325. doi: 10.3389/fimmu.2022.961325 36217542 PMC9547048

[161] Lee HB Lee J Jeong K Yang J Jung YH Moon JS . Randomized, double-blind, placebo-controlled trial of ID-CBT5101, a tyndallized Clostridium butyricum postbiotic, in adults with mild-to-moderate knee osteoarthritis. J Microbiol Biotechnol. (2026) 36:e2512024. doi: 10.4014/jmb.2512.12024 41581926 PMC12861722

[162] Marco ML Sanders ME Ganzle M Arrieta MC Cotter PD De Vuyst L . The International Scientific Association for Probiotics and Prebiotics (ISAPP) consensus statement on fermented foods. Nat Rev Gastroenterol Hepatol. (2021) 18:196–208. doi: 10.1038/s41575-020-00390-5 33398112 PMC7925329

[163] Melendez-Oliva E Martinez-Pozas O Sinatti P Martin Carreras-Presas C Cuenca-Zaldivar JN Turroni S . Relationship between the gut microbiome, tryptophan-derived metabolites, and osteoarthritis-related pain: A systematic review with meta-analysis. Nutrients. (2025) 17:264. doi: 10.3390/nu17020264 39861394 PMC11767305

[164] Niu L Chen W Yin Z Tan H Cui J Su J . Bacterial extracellular vesicles in osteoarthritis: A new bridge of the gut-joint axis. Gut Microbes. (2025) 17:2489069. doi: 10.1080/19490976.2025.2489069 40213946 PMC12716045

[165] Demehri S Kasaeian A Roemer FW Guermazi A . Osteoarthritis year in review 2022: Imaging. Osteoarthritis Cartilage. (2023) 31:1003–11. doi: 10.1016/j.joca.2023.03.005 36924919 PMC12224773

[166] Mohammadi S Salehi MA Jahanshahi A Shahrabi Farahani M Zakavi SS Behrouzieh S . Artificial intelligence in osteoarthritis detection: A systematic review and meta-analysis. Osteoarthritis Cartilage. (2024) 32:241–53. doi: 10.1016/j.joca.2023.09.011 37863421

[167] Diniz P Grimm B Garcia F Fayad J Ley C Mouton C . Digital twin systems for musculoskeletal applications: A current concepts review. Knee Surg Sports Traumatol Arthrosc. (2025) 33:1892–910. doi: 10.1002/ksa.12627 39989345

[168] Ma J Chen C Jiang H Xi M Luo W Wan R . Mechanisms and precision interventions in sarcopenia and osteoarthritis comorbidity: A narrative review. J Orthop Translat. (2026) 58:101093. doi: 10.1016/j.jot.2026.101093 42006907 PMC13085027

[169] Zhu Q Qi N Shen L Lo CC Xu M Duan Q . Sexual dimorphism in lipid metabolism and gut microbiota in mice fed a high-fat diet. Nutrients. (2023) 15:2175. doi: 10.3390/nu15092175 37432375 PMC10180580

[170] Alsegiani AS Shah ZA . The influence of gut microbiota alteration on age-related neuroinflammation and cognitive decline. Neural Regener Res. (2022) 17:2407–12. doi: 10.4103/1673-5374.335837 35535879 PMC9120705

[171] Costa CM Pedrosa SS Kirkland JL Reis F Madureira AR . The senotherapeutic potential of phytochemicals for age-related intestinal disease. Ageing Res Rev. (2025) 104:102619. doi: 10.1016/j.arr.2024.102619 39638096

[172] Peron G Gargari G Merono T Minarro A Lozano EV Escuder PC . Crosstalk among intestinal barrier, gut microbiota and serum metabolome after a polyphenol-rich diet in older subjects with "leaky gut": The MaPLE trial. Clin Nutr. (2021) 40:5288–97. doi: 10.1016/j.clnu.2021.08.027 34534897

[173] Cai J Chen Z Wu W Lin Q Liang Y . High animal protein diet and gut microbiota in human health. Crit Rev Food Sci Nutr. (2022) 62:6225–37. doi: 10.1080/10408398.2021.1898336 33724115

[174] Amin N Liu J Bonnechere B MahmoudianDehkordi S Arnold M Batra R . Interplay of metabolome and gut microbiome in individuals with major depressive disorder vs control individuals. JAMA Psychiatry. (2023) 80:597–609. doi: 10.1001/jamapsychiatry.2023.0685 37074710 PMC10116384

[175] Wang Q Dai H Hou T Hou Y Wang T Lin H . Dissecting causal relationships between gut microbiota, blood metabolites, and stroke: A Mendelian randomization study. J Stroke. (2023) 25:350–60. doi: 10.5853/jos.2023.00381 37813672 PMC10574297

[176] Angelini F Widera P Mobasheri A Blair J Struglics A Uebelhoer M . Osteoarthritis endotype discovery via clustering of biochemical marker data. Ann Rheum Dis. (2022) 81:666–75. doi: 10.1136/annrheumdis-2021-221763 35246457

[177] Zaki S Blaker CL Little CB . OA foundations - experimental models of osteoarthritis. Osteoarthritis Cartilage. (2022) 30:357–80. doi: 10.1016/j.joca.2021.03.024 34536528

[178] Batushansky A Zhu S Komaravolu RK South S Mehta-D'souza P Griffin TM . Fundamentals of OA. An initiative of Osteoarthritis and Cartilage. Obesity and metabolic factors in OA. Osteoarthritis Cartilage. (2022) 30:501–15. doi: 10.1016/j.joca.2021.06.013 34537381 PMC8926936

[179] Mamic P Snyder M Tang WHW . Gut microbiome-based management of patients with heart failure: JACC review topic of the week. J Am Coll Cardiol. (2023) 81:1729–39. doi: 10.1016/j.jacc.2023.02.045 37100490

[180] Sadee W Wang D Hartmann K Toland AE . Pharmacogenomics: Driving personalized medicine. Pharmacol Rev. (2023) 75:789–814. doi: 10.1124/pharmrev.122.000810 36927888 PMC10289244

[181] Bian M Zhu C Nie A Zhou Z . Guizhi Shaoyao Zhimu decoction ameliorates gouty arthritis in rats via altering gut microbiota and improving metabolic profile. Phytomedicine. (2024) 131:155800. doi: 10.1016/j.phymed.2024.155800 38851098

